# Epoxy-Functional (Alkyl)methacrylate-Based Hybrids Reinforced with Layered Silicate Montmorillonite: From Mechanistic Study to Sustainable Wastewater Treatment

**DOI:** 10.3390/gels11100803

**Published:** 2025-10-07

**Authors:** Berran Sanay, Rabia Bozbay, Sena Ciftbudak, Zeynep Ulker, Sevval Teke, Zuhal Akyol, Elif Pelin Ozdemir, Nermin Orakdogen

**Affiliations:** 1 Department of Chemistry, Graduate School of Science Engineering and Technology, Istanbul Technical University, Maslak, 34469 Istanbul, Turkey; sanay15@itu.edu.tr (B.S.); bozbay19@itu.edu.tr (R.B.); ciftbudak@itu.edu.tr (S.C.); ulkerz19@itu.edu.tr (Z.U.); tekes20@itu.edu.tr (S.T.); akyolz21@itu.edu.tr (Z.A.); 2 Department of Chemistry, Faculty of Science and Letters, Istanbul Technical University, Maslak, 34469 Istanbul, Turkey; ozdemirel22@itu.edu.tr

**Keywords:** montmorillonite, hybrid, crosslinking, elasticity, swelling, adsorption

## Abstract

This work aims to design versatile hybrids fabricated by poly(hydroxypropyl methacrylate-co-glycidyl methacrylate) gels loaded with pristine montmorillonite, P(HPMA-co-GMA)/Mmt, by varying the clay content. Insights into design of epoxy-functional hybrids were provided by combining in situ copolymerization reactions with solution mixing to evaluate the effect of aluminosilicate addition on structure–property changes in (alkyl)methacrylate-based gels. Comprehensive analyses were conducted regarding the composition and structural properties of hybrids in the presence of Mmt. The hybrids exhibited excellent swelling, salt surfactant tolerance, and pH sensitivity depending on the composition. The higher the Mmt concentration, the lower the swelling ratio; however, the compressive moduli did not change monotonically with increasing Mmt from 0.80 to 2.20% (*w*/*v*). Dye adsorption revealed the effects of variables (dye type, pH, contact time, concentration) on adsorptive properties of hybrids towards cationic methylene blue (MB) and anionic sunset yellow, allura red, blue brilliant, carmoisine, and tartrazine dyes. Adsorption kinetics of MB obeyed pseudo-second-order model, and the maximum dye adsorption capacity for hybrids increased from 5.01 mg g^−1^ to 16.42 mg g^−1^, while adsorption isotherms were defined by the Freundlich model. The proposed hybrids have emerged as alternative materials that enable multiple uses of same adsorbent for the removal of different types of pollutants.

## 1. Introduction

Epoxy-functional clay nanocomposites have been shown to improve structural properties and mechanical behavior, depending on the type of clay modifier and the dispersion method [1,2,3]. In nanoclay-filled polymer-based systems, generally, tensile modulus and fracture toughness increase with increasing clay loading, and the chemical structure of nanocomposites becomes important in improving the tensile strength [4]. Despite its appealing characteristics for the production of filled polymeric materials, (alkyl)methacrylate-based gels have considerable mechanical limitations. The extent of the interactions and thermodynamical forces generated due to the thermal motion of the chains in the polymer network, constrained by covalent or physical crosslinks, determines the overall gel elasticity.

Since the elasticity of a crosslinked polymer network emerges as the most important macroscopic property, understanding how the properties of polymer network, the functionality of crosslinkers, and the magnitude of microscopic interactions between chain segments determine the elastic response is crucial for the material design [5]. The elastic modulus, which varies depending on the crosslinking density and the chain length distribution for networks with the same crosslinker concentrations, is controlled by the density of components and is highly dependent on the network design protocol. In real systems, it is difficult to understand how many different elements, such as the macroscopic behavior of the network, the type and properties of the organic/inorganic components, the synthesis protocol followed and the thermodynamic parameters, contribute to the elastic properties of the material [6]. The aluminosilicate layers of smectite clays such as montmorillonite (Mmt) contain extremely hard flakes with a very high aspect ratio, 1 nm thick and 100 to 300 nm in diameter. As the clay minerals are so hard, estimated to be 160–400 GPa, the stress transfer from the polymer to the mineral increases the strength of network, while the polymer-based structure imparts flexibility to the material, preventing brittleness [7]. In clay–polymer nanocomposites, where the clay is less than 5% wt, ideally the complete exfoliation of clay layers, coupled with their homogeneous distribution throughout the polymer matrix due to their random orientation, improves the overall material properties.

To achieve the targeted properties, the distribution of the clay layers within the polymer-based structure must be controlled at the microscale [8]. Innovative synthetic approaches have been developed for exfoliating clay layers, separating them, and regulating the interaction energy between the polymer network and these layers. The lack of topological studies to find suitable clay and polymer combinations, synthesis routes, and optimal clay/polymer concentrations are limitations to the production of clay–polymer nanocomposites and potential large-scale engineering [9]. Studies are needed to adjust the effective distribution of clay layers by reducing the free energy required to separate these layers. In the presence of inorganic clay, most of the polymer remains at the interface and interacts with the clay surface. In this structure, polymer–clay interactions become important as a majority of the polymer is at the interface, changing its properties compared to the bulk state [10]. The exfoliation of clay structures depends on the elastic forces generated during the polymerization. The crosslinked polymer chains, with increasing molecular weight as the polymerization proceeds, store more energy in terms of the storage modulus [8]. However, van der Waals forces and the electrostatic attractions between adjacent clay layers, which hold the growing chains within them, prevent the crosslinked structure from recoiling. As a result, conformational entropy, which is the result of the ability of the crosslinked polymer chains to relax, gradually increases until a critical point where the attractive forces and the entropic elastic forces are balanced. The dominance of elastic forces over attractive forces pushes adjacent clay layers apart [11]. Considering all the forces acting on adjacent clay layers, the elastic force resulting from the conformational entropy works to separate the clay layer, while the sum of the attractive and viscous forces resulting from the van der Waals forces and electrostatic attraction work against the exfoliation. When the elastic forces are more dominant, the exfoliation of the clay occurs, while the faster the viscosity of the pre-gel solution increases, the harder it is to exfoliate the clay layer. The process of chain relaxation during polymerization and the possibility of the formation of exfoliated structures by pushing the clay layers out of the tactoids are largely determined by the change in the viscosity of macromolecules in the extragallery with time. Based on the differences in polymerization rates, Park and Jana showed that the diffusion of molecules and crosslinkers into the galleries occurs more easily in the lower viscosity medium, since the lower viscosity gelation medium facilitates the exfoliation [11].

Since this layered structure allows for precise adjustments and enables the design of effective composite materials, Mmt nanoclay, with its high adsorption capacity, has gained importance as a versatile clay mineral, particularly in wastewater treatment and combating water pollution. Mmt nanoclay was widely employed as an adsorbent to remove the cationic and anionic dyes simultaneously by Zhang and coworkers [12]. In their work, cationic dye methyl violet, and anionic dyes methyl orange and naphthol green B were treated by Mmt clay. The molecular arrangements of dyes in Mmt interlayer were analyzed and the molecular dynamics simulation of dyes entering the interlayers was presented. Based on the continuous development of sustainable solutions for water pollution by further improving the properties of composite adsorbents, Mmt-based adsorbents are increasingly preferred in practical applications due to their effectiveness and environmental friendliness. To study the effect of clay content, Çavuşoğlu and Kasgoz prepared poly(2-hydroxyethyl methacrylate)/Mmt nanocomposites using free radical polymerization by varying the Mmt content between 3 and 20% wt [13]. While the exfoliation was observed in the Mmt layers for the nanocomposites obtained with lower clay addition, at higher clay ratios, the interlayer clay structure continued to exist along with the exfoliation structure. Based on the ability to obtain different structures by varying the preparation conditions, Essawy and coworkers studied poly(methyl methacrylate)/Mmt nanocomposites synthesized via in situ intercalative suspension and emulsion polymerization by varying the initiator conditions during polymerization [14]. At low initiator concentrations, a weakly intercalated structure was achieved, while higher initiator concentrations resulted in the exfoliated nanocomposites. Djouani and coworkers prepared montmorillonite/poly(glycidyl methacrylate) (Mmt/PGMA) nanocomposites via in situ atom transfer radical polymerization resulting in a highly exfoliated structure [15]. By dispersing Mmt/PGMA nanofiller in epoxy resin, ternary composites with excellent dispersion were synthesized due to the increased compatibility of PGMA chains with the resin as a result of the presence of epoxy groups in the structure. Compared to pure crosslinked epoxy adhesives without clay, ternary systems with superior viscoelastic properties and storage modulus were obtained. Recently, sulfonated poly(glycidyl methacrylate-co-styrene) copolymers have emerged as functional adsorbents that can be used for the selective removal of methylene blue from aqueous solutions [16]. The results showed that epoxy-functional polymers can be applied as multipurpose polymeric materials with a wide range of industrial uses.

In order to protect aquatic ecosystems, amine-functionalized PGMA gels were used for the adsorption of Congo red and are shown to have significant potential for the effective adsorption of anionic dyes due to the presence of different number and length of amino groups [17]. Lapwanit and coworkers proposed a feasible strategy for the removal of cationic methylene blue by the adsorption of environmentally friendly κ-carrageenan/PGMA beads by incorporating PGMA units with reactive epoxide functional groups into the κ-carrageenan matrix [18]. The efficacy of the combination of the electron-rich structure of PGMA with anionic functionality of κ-Carrageenan against cationic dyes has been studied. Different blend compositions, which contain reactive epoxy groups in the PGMA long molecular chain and enables the material to interact with cationic dyes, followed the pseudo-second-order model and fits well with the Langmuir isotherm, with a maximum methylene blue adsorption of 166.62 mg g^−1^. The repeating units in the structure were designed to contain reactive sulfate and epoxide functional groups that are suitable for binding with the cationic dye via electrostatic mechanism. In another work, Elwakeel and coworkers showed efficient removal of Reactive Black 5 (RB5) from aqueous media using glycidyl methacrylate/methylenebisacrylamide resin modified with tetraethelenepentamine [19]. In the sorption process based on the anion exchange mechanism with the presence of amino groups in the resin structure, approximately 90% of the total RB5 uptake was achieved within 60 min. In natural fiber processing technology, the combination of Mmt with GMA-modified Kenaf bast fiber has been shown to impact the performance of nanofiller-doped polymer composites by affecting the stress transfer efficiency between the polymer matrix and the filler [20]. The reactive sites on the Mmt surface resulting from chemical modification with GMA enhance the crosslinking between the matrix and Mmt nanoclay, improving the interfacial adhesion and enhancing the compatibility between the polyester matrix and nanoclay.

Within the scope of the presented literature, varying amounts of the inorganic nanoclay Mmt were integrated into the functionalized copolymer structure consisting of glycidyl methacrylate (GMA) and hydroxypropyl methacrylate (HPMA) to form an effective (alkyl)methacrylate-based hybrid structure. Based on the combination of nanoclay Mmt and epoxy functionalization, research effort is focused on developing a functionalized hybrid structure that acts as an alternative effective adsorbent for the selective removal of anionic and cationic dyes from aqueous solutions at low production cost. The study hypothesized that in addition to the functional properties of the aluminosilicate Mmt, combining the epoxy-functional monomer GMA with the methacrylic acid ester HPMA could lead to the development of versatile hybrid poly(hydroxypropylmethacrylate-co-glycidylmethacrylate)/montmorillonite structures suitable for a variety of industrial applications. In the synthetic strategy employed, the pendant epoxy group in the structure could be used as a precursor to a multifunctional hybrid to react with nucleophiles to obtain multipurpose polymeric materials with additional functionalities or reactive properties. Accordingly, GMA monomer is widely preferred in the preparation of various composites due to its advantage of providing epoxy functionalization to other acrylate monomers. As an ester of methacrylic acid and glycidol, GMA enables the synthesis of functional composites by providing epoxy functionality, while HPMA was chosen as it is an economical synthetic component with strong copolymer formation ability and good dimensional stability. Another advantage is that GMA, a highly reactive, hydrophobic monomer with dual functionality of methacrylic and epoxy groups, readily copolymerizes with other monomers to form advanced materials. The pendant epoxy group in the desired structure can serve as a precursor for multifunctional polymers with additional functionalities or reactive properties. With the combination of these properties, a perspective is presented on how Mmt integration changes the overall elastic properties based on its effect on the degree of swelling. The adsorption properties of resultant (alkyl)methacrylate-based hybrids for the removal of cationic and anionic dyes were analyzed by performing bulk adsorption studies by varying the operational parameters: dye type and concentration, contact time, and pH of the medium.

## 2. Results and Discussion

Innovative copolymeric poly(hydroxypropyl methacrylate-co-glycidyl methacrylate)/Montmorillonite (PHG/Mmt) hybrids containing functional epoxide and hydroxyl groups were synthesized by varying the aluminosilicate content in the hybrid structure. A perspective was established by examining the physicochemical properties of hybrids with nanoclay-integrated properties and their application as adsorbents in the treatment of dye wastewater.

### 2.1. Synthesis of Epoxy-Functional (Alkyl)methacrylate-Based Hybrid Gels

**Scheme 1:**
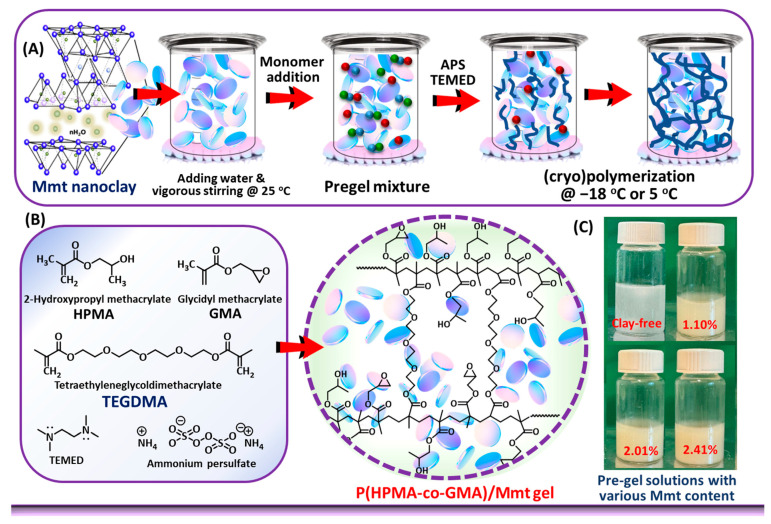
(**A**) Illustration of the synthetic protocol followed for the preparation of Mmt-integrated hybrid gels. (**B**) Structure of the components used in the preparation of hybrid P(HPMA-co-GMA)/Mmt gels. (**C**) Optical views of the pre-gel solutions for the clay-free blank PHG gel and the solutions containing various amount of Mmt just before the addition of monomers.

The formation of layered silicate-integrated epoxy-functional hybrid gels was achieved via the combination of in situ free-radicalic crosslinking copolymerization reaction with the blending method according to Scheme 1. The concentration of nanoclay Mmt integrated into the copolymer poly(hydroxypropyl methacrylate-co-glycidyl methacrylate) network, in which the mol ratio of HPMA/GMA monomers was fixed as 80/20%, varied between 0 and 2.41% (*w*/*v*). The total monomer concentration in the feed for HPMA, GMA, and TEGDMA in the structure was set to 15.1% (*w*/*v*). In the synthetic protocol followed for the chemical crosslinking of the copolymer, the crosslinking ratio X, the mole ratio of TEGDMA to HPMA and GMA was fixed at 1/78 [21]. Table S1 presents the composition of epoxy-functional hybrid gels prepared at various Mmt contents. Optical views of the clay solutions containing various Mmt just before the addition of monomer was also presented in Scheme 1. For the clay-free blank copolymer synthesis, coded as PHG/Mmt0, the pre-gel solution was whitish opaque, and as Mmt content increased up to 2.41% (*w*/*v*), the yellowish opacity of the solutions also increased.

**Figure 1:**
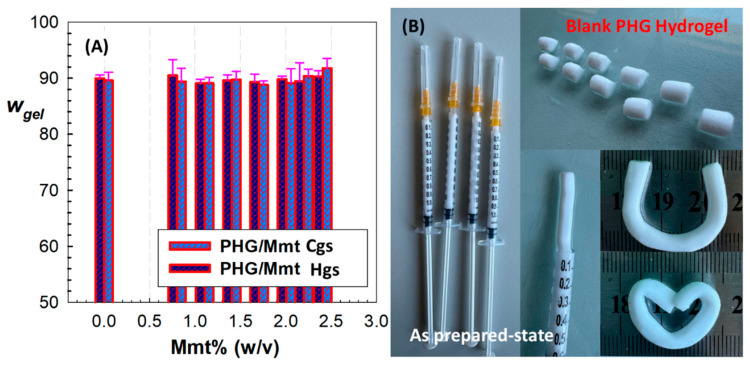
Gel fraction of hybrid PHG/Mmt hydrogels and cryogels as a function of Mmt concentration (**A**), as-prepared state optical images of blank PHG/Mmt hydrogel sample (**B**).

Table 1 shows the changes in the characteristic network parameters for hybrid hydrogels and cryogels depending on the amount of nanoclay Mmt in the hybrid composition. Upon completion of the crosslinking polymerization to remove the impurities by a water solvent exchange procedure, the cylindrical gel rods removed from the syringes were subjected to the swelling/drying stages to determine the structural parameters that characterize the network structure [22]. Using the dry gel weights, the characteristic experimental values of $\nu_{2}^{0}$, defined as the volume fraction of the crosslinked polymer network after the preparation stage, and the change in gel fraction as a function of the Mmt content in the hybrid structure were calculated using Equation (1). The difference between the calculated theoretical values and the experimental values, depending on the synthesis conditions, was attributed to the presence of bound water remaining in the gel structure during the drying process. The high agreement between the theoretical and experimental values for hybrid hydrogels and cryogels, depending on the Mmt concentration, indicates the success of the synthesis. In Figure 1, depending on the Mmt concentration, the gel fraction of hybrid PHG/Mmt hydrogels and cryogels varied between 89.96 (±0.61)–90.30 (±1.03) and 89.58 (±1.50)–91.77 (±1.75), respectively. Functional epoxy groups were introduced into the PHPMA structure by copolymerizing HPMA with GMA carrying an epoxy group. Table 1 and the gel fraction values provide accurate data on free radical crosslinking copolymerization of PHG/Mmt gels over the entire range for Mmt concentrations. From Figure 1, it is clear that free-radicalic polymerization produces high-yield hybrid networks, depending on the amount of clay used. This results in a simplification of the functionalization step, as the oxirane group in the GMA structure serves as an active site for effective covalent binding in the polymerization reaction.

In terms of polymerization kinetics, the studies have shown that GMA units in the structure have a faster polymerization rate than hydroxyethyl methacrylate (HEMA) [23], as the HEMA units of hybrid structure provide the hydrophilicity of copolymer network while controlling the density of epoxy groups. The contribution of HPMA units to polymerization is due to the methacrylate groups in its structure, which lead to relatively easy radical reactions, while hydroxyl groups provide the hydrophilicity [24]. In the hybrid structure, the carbonyl C=O group of HPMA monomer functions as a proton acceptor, while the hydroxyl –OH unit functions as both a proton donor and acceptor. Therefore, the hydrogen bond formation between the carbonyl oxygen atom and the hydroxyl group in the structure strengthens the partial charges on the carbonyl C atom, creating charge transfer. Both C=O···HO and OH···-type H-bonds can form between PHPMA units in the hybrid structure, with the possible contribution of the carbonyl units of GMA. For the H-bond formation in PHEMA, as a result of the analysis by temperature-dependent infrared spectroscopy, it was reported that 47.3% of the OH group in the terminal side chain of PHEMA participated in the OH···O=C hydrogen bond formation, while 53.7% contributed to the OH···OH hydrogen bond formation [25]. The addition of Mmt platelets to the polymerization medium disrupts the HPMA-HPMA and HPMA-GMA interactions, creating more reactive molecules that increase the reaction rate during the polymerization. This results in higher kinetic rate constants and lower overall activation energies. Thus, preventing the formation of potential H-bonds between HPMA and GMA relatively increases the rate of monomer addition to the macroradicals. Because the preparation temperature of PHG/Mmt hydrogels synthesized at room temperature is higher, the formation of side H-bonds is not dominant, as monomer molecules and macroradicals have greater mobility. However, since the reactivity of radicals and monomer molecules decreases significantly at lower temperatures due to H-bond formation, the presence of Mmt platelets, which disrupt these bonds, will significantly contribute to the increased polymerization rate.

Ren and coworkers applied surface-initiated atom transfer radical polymerization to prepare poly(2-hydroxyethyl methacrylate)-block-poly(2-hydroxyethyl methacrylate-co-glycidyl methacrylate) brushes and followed the polymerization kinetics using quartz crystal microbalance [23]. To control the density of epoxy groups, two GMA/HEMA ratios, 1:10 and 1:4, were examined in terms of the feed ratio between GMA and HEMA, and the results of bulk polymerization kinetics suggested the faster polymerization rate of GMA than HEMA. Zhao and coworkers reported the design of poly(hydroxyethyl methacrylate-co-glycidyl methacrylate)-grafted magnetic chitosan microspheres via reversed-phase suspension polymerization. The grafted GMA and HEMA were reported to reduce steric hindrance during binding by providing reactive epoxy groups, thus increasing enzyme immobilization capacity and preserving the activity by creating a hydrophilic microenvironment on the surface [26]. The slight increase in the polymerization rate caused by Mmt integration into PHEMA was attributed to non-covalent interactions such as hydrogen bonding between the polymer and the nanoadditive in the medium. Achilias and Siafaka analyzed the formation of PHEMA-based nanocomposites via following in situ bulk radical polymerization kinetics in the presence of organomodified Mmt [27]. The effect of nanofiller content on the double bond conversion and hence the polymerization rate over time at two different temperatures was investigated. The presence of nanoclay increased the polymerization kinetics, leading to higher conversion and the relative effective rate constant increased significantly with the amount of Mmt but showed a much lower increase at higher temperatures.

### 2.2. Structural Characterization of Epoxy-Functional Hybrid Gels

**Figure 2:**
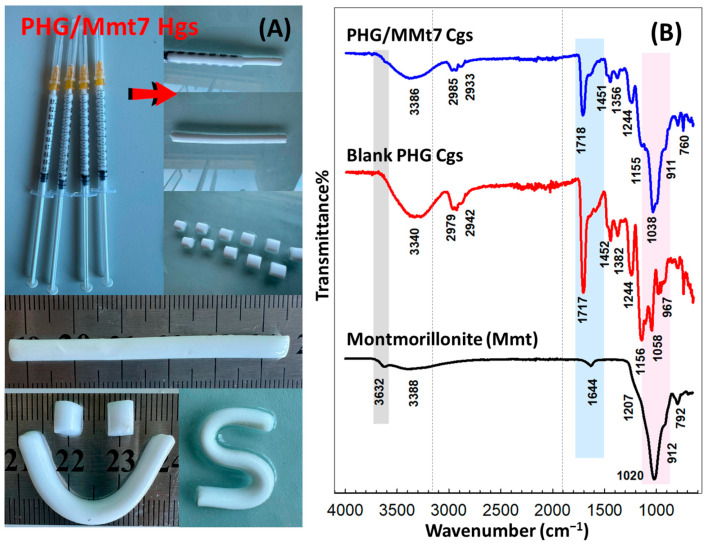
(**A**) Optical images of the hybrid sample at the completion of synthesis, removal from the syringe and preparation of cylindrical samples for applications. (**B**) ATR-FTIR spectra of raw Mmt, blank PHG and hybrid PHG/Mmt7 cryogel containing 2.41% (*w*/*v)* Mmt in the feed. Different colors represent the functional groups in the hybrid structures.

Figure 2 presents ATR-FTIR spectra of Mmt clay, blank PHG as a reference gel, and hybrid PHG/Mmt7 cryogel containing 2.41% (*w*/*v*) Mmt in the feed. For comparison, ATR-FTIR spectra of hybrid hydrogels are presented in Figure S1 in Supplementary Materials. The broad bands observed at 3340 and 3386 cm^−1^ in the spectra of blank PHG and hybrid PHG/Mmt7 cryogel were attributed to the stretching vibration of lateral hydroxyl groups, -OH-belonging HPMA units. The stretching vibration peaks detected at around 2979 and 2942 cm^−1^ for blank PHG and at 2985 and 2933 cm^−1^ for hybrid gels were attributed to -CH_2_-, C-H, and -CH_3_ bond stretching. For blank PHG gel, the absorption peak observed at approximately 1717 cm^−1^ was due to the stretching vibration of the carbonyl groups, whereas after the addition of Mmt, the peak intensity at 1718 cm^−1^ decreased due to the increased hydrogen bond interaction [28]. For the blank PHG Cgs, the peak appearing at 1244 cm^−1^ was detected as belonging to the C–O–C bond of the epoxy group, with decreasing intensity for the hybrid PHG/Mmt7 Cgs. The peaks at 1155–1156 cm^−1^ attributed to -C-O- stretching vibration indicated the presence of -COO- ester groups in the prepared cryogels. The peak at 967 cm^−1^ corresponding to the epoxy group of GMA for the blank PHG gel was detected as a shoulder around 964 cm^−1^ in the hybrid PHG/Mmt7 Cgs spectrum.

In the spectrum of raw Mmt, the peak at 3632 cm^−1^ was attributed to the presence of hydroxyl linkage, while the strong band at 3388 cm^−1^ and the overlaid absorption peak at 1644 cm^−1^ predicted the possibility of hydration of water or -OH bending mode of adsorbed water. The shoulder at 1207 cm^−1^ was attributed to the bending mode of adsorbed water by Mmt, while the strong band at 1020 cm^−1^ was observed due to Si–O–Si stretching (in plane) vibration for layered silicates. In the hybrid structure, the shoulder at 911 cm^−1^ corresponded to the bending mode of -OH groups of Mmt via Mg-Al-OH linkage, and the shift in the peaks suggested that exchangeable Na^+^ tends to settle preferentially into ditrigonal cavities over sites of ionic substitution through the octahedral sheet [29]. The peaks in the range of about 760–790 cm^−1^ in the hybrid spectrum are due to the stretching of the Mg-Fe-OH and Si-O-Si tetrahedral bridge bonds in SiO_2_. Discrete differences observed in the shift and intensity of the absorption bands in the spectrum of hybrid compared with the clay-free copolymeric structure were a consequence of incorporating inorganic nanoclay Mmt as well as the crosslinking reactions. All these peaks confirmed that clay-free copolymeric as well as (alkyl)methacrylate-based hybrid gels have been prepared successfully.

### 2.3. Thermogravimetric Analysis of Epoxy-Functional Hybrid Gels

**Figure 3:**
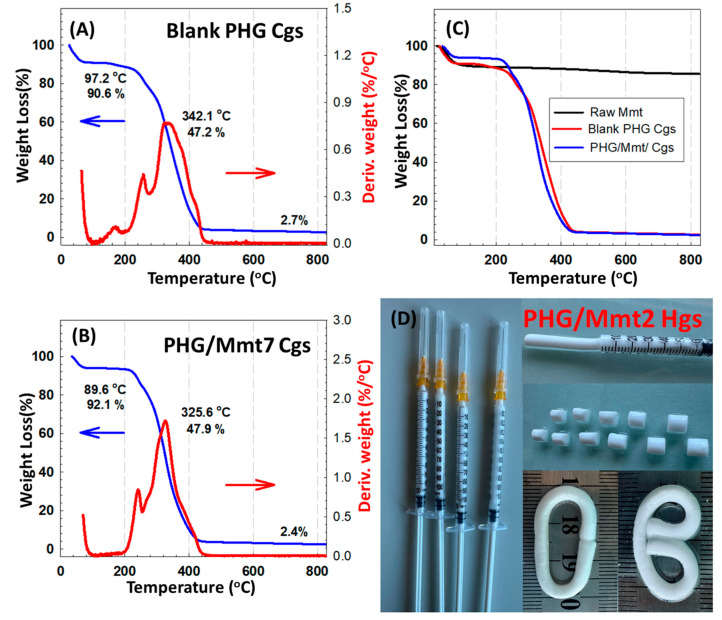
Thermograms (TGA) and differential thermogravimetry (DTG) curves of clay-free blank PHG Cgs (**A**), that of hybrid PHG/Mmt7 Cgs (**B**), and the comparison of TGA curves of the thermal decomposition with raw Mmt (**C**). Optical images of the hybrid sample at the completion of synthesis, removal from the syringe, and preparation of cylindrical samples for applications (**D**).

After the cylindrical gels were subjected to the solvent-exchange procedure in water to remove the impurities, they were dried and ground into powder to perform the thermogravimetric analysis for blank PHG and hybrid PHG/Mmt gels. The obtained thermograms were compared and discussed in terms of the changes observed in the co- and hybrid network structures resulting from the interactions between the functional groups and inorganic clay Mmt. In Figure 3, the thermal curve for raw Mmt indicated the loss of interlayer water with 3.04% to about 112.4 °C, in which the mass loss reached its maximum at 51.5 °C [30]. At 205 °C, the slope of curve started to decrease slightly through 600 °C, after which only 13.7% of water associated with the exchangeable cation was lost, and above 600 °C, a very small amount of water was evolved, namely 0.5%. The mass loss process in all samples takes place in two steps. For hydrogel samples of blank PHG and hybrid PHG/Mmt given in Figure S2, the first step observed up to 202.8 and 208.2 °C with a mass loss of 9.6% and 7.4% due to the physical desorption of water from the surface, respectively. In the first region, the mass loss of cryogel samples of blank PHG and hybrid PHG/Mmt Cgs were 10.9% and 6.6% below 184.9 and 189.5 °C, respectively. For copolymeric PHG Hgs and Cgs, following the initial water loss, the second stage occurred in the range of 202.8–440.8 °C and 184.9–449.1 °C, respectively, with a mass loss of 95% and identical peak temperatures at 342 °C. The second decomposition region of hybrid PHG/Mmt Hgs was in the range of 208.2 and 450.5 °C with a corresponding peak temperature at 368.2 °C and mass loss of 94.9%. While for hybrid PHG/Mmt Cgs, the second decomposition region was observed between 189.5 and 453.1 °C with a mass loss of 96.2% and peak temperature at 325.6 °C, which was due to the thermal decomposition of side groups in polymer chains, the elimination of chain-end monomers, as well as the ester side-chain scissions. Moreover, a weak broad peak was observed for PHG Hgs and Cgs in the range of 210–270 °C as well as for hybrid PHG/Mmt Hgs and Cgs in the range of 180–260 °C due to the degradation of epoxy C–O–C groups in GMA units. Similar results were reported by Zhai and coworkers for P(HPMA-co-GMA) cryogels prepared in the presence of *N,N*-methylenebisacrylamide as the crosslinker [31]. All the hybrid gels synthesized in this study were thermally more stable than blank PHG gels according to the decomposition temperature as well as the residue at 800 °C. Table 2 shows the thermogravimetric results collected and compared with blank PHG and hybrid PHG/Mmt Hgs and Cgs. The first derivative of the mass losses of all prepared Hgs and Cgs with respect to temperature showed the location of the maximum temperature Tmax, which increased with the addition of Mmt nanoclay. The hydroxyl group of PHPMA is able to interact with the GMA units, and the resulting interactions are responsible for the position of these maximum peaks. Depending on both the changing polymerization temperature and Mmt concentration, the residue of hydrogel samples at 800 °C was 1.4% and 3.6% for blank PHG and hybrid PHG/Mmt, respectively, while the residue of cryogel samples was 2.7% and 2.4%.

Tang and colleagues recently reported that Mmt nanoclay integrates into the network, acting as a barrier to hydrogel mobility and thus increasing the thermal stability. Therefore, poly(2-acrylamide-2-methylpropane sulfonic acid)/polyethylene glycol/montmorillonite (PAMPS/PEG/Mmt) hydrogels exhibited slower weight loss compared to the reference gel without clay. This suggests that the addition of Mmt increases the thermal stability of the hydrogels through the interactions between the ionic functional groups of Mmt and PAMPS [32]. Jumpapaeng and coworkers prepared cassava starch-grafted polyacrylamide/natural rubber nanocomposites containing various Mmt contents (0–10 wt%) to investigate the effect of nanoclay addition on the thermal stability [33]. The gradual increase in thermal stability with increasing amount of Mmt nanoclay was due to the formation of stronger physical crosslinks between the polymeric networks by acting as crosslinking agents of the -OH groups on Mmt.

**Figure 4:**
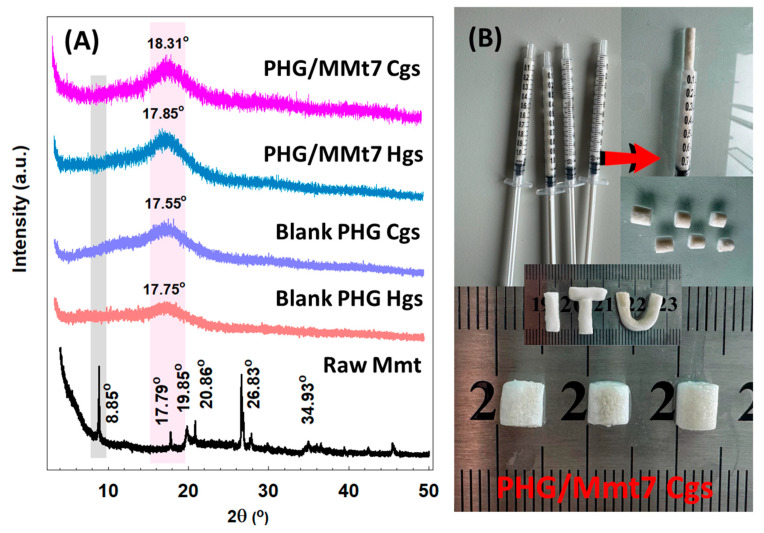
XRD patterns of raw Mmt, blank PHG, and Mmt-integrated hybrid PHG/Mmt hydrogel and cryogels (**A**), optical images of 2.41% (*w*/*v*) Mmt-doped cryogel samples as-prepared state (**B**). The red arrow indicates the preparation of small cylindrical gel samples for swelling and elastic modulus measurements after removing the gel rods from the polypropylene syringe.

XRD patterns of blank PHG as well as hybrid PHG/Mmt7 Hgs and Cgs are shown in Figure 4. The interlayer spacing calculated from the reflection peaks according to Bragg’s equation; d = nλ/2sin θ, predicted that Mmt exhibits a characteristic reflection at 2θ 8.85°, which corresponds to a basal spacing (d_001_) of about 9.9562 Å. The other peaks at 2θ 17.81° and 19.83° are impurities like quartz, which was further confirmed by the characteristic band at 792 cm^−1^ in the ATR-FTIR spectrum of Mmt, and the presence of kaolinite was indicated by d-spacing peaks at 20.86° and 26.83° and the small broader shoulder around 34.93° [34]. It is clear from the sharpness and intensity of the peaks in the spectrum that Mmt has a highly crystalline structure. However, the reflection of Mmt at 2θ 8.85° seemed to be diminished for hybrid PHG/Mmt7 Hgs and Cgs which exhibited an exfoliated structure. For copolymeric and hybrid hydrogel and cryogel samples, there was a marked similarity in the diffractograms in which the broad and low intensity bands indicated a low degree of crystallinity as well as a small crystallite size which is consistent with previously published data [35,36].

At 2θ degrees of around 17.85° and 18.31°, the broad and less intense bands were detected for the hybrid PHG/Mmt7 samples; however, similar bands with smaller intensity indicating the semi-crystalline regions were detected at 2θ = 7.75°, and 17.55° for the copolymer PHG Hgs and Cgs, respectively. A major X-ray diffraction peak at around 20.5° showing the crystalline region was recently reported by Yang and coworkers for HPMA grafted onto the surface of silk fabric to improve its crease resistance [36]. Consequently, XRD results confirmed the formation of nanocomposites successfully by the exfoliation of Mmt where the layers were distributed unevenly between the network chains without interrupting the formation of crystalline regions. The polar groups in the blank and hybrid PHG/Mmt7 gels interact with the polar groups of Mmt clay, and the corresponding interaction results in the production of stronger and denser networks.

### 2.4. Elastic Properties of Epoxy-Functional Hybrid Gels

**Figure 5:**
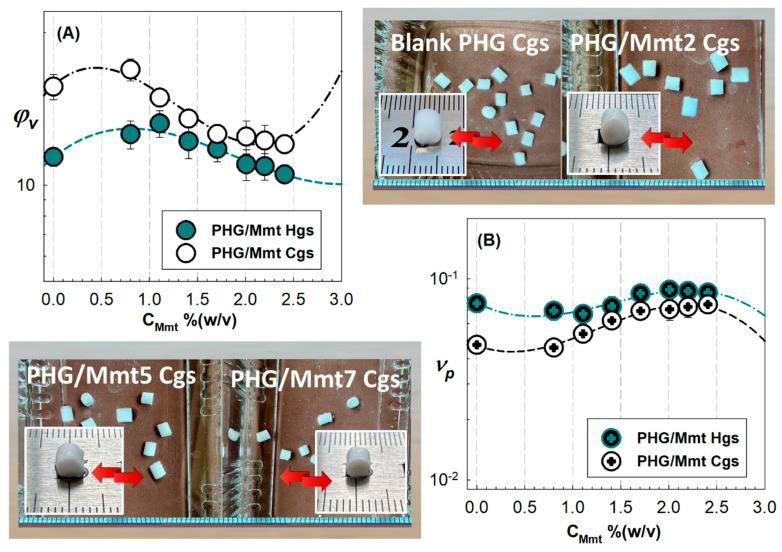
(**A**) The equilibrium swelling ratio of Mmt-doped PHG/Mmt Hgs and Cgs in water and (**B**) the volume fraction of the crosslinked polymer network in the swollen hybrid gels plotted against the Mmt concentration. The values are mean ± SD, *n* = 4. Error bars are not visible if they are the same size or smaller than the symbols.

Figure 5 shows the equilibrium swelling ratio of Mmt-doped PHG/Mmt gels in water and the volume fraction of the crosslinked polymer network in the swollen hybrids as a function of Mmt concentration. Considering that HPMA is a more polar component than GMA units, the HPMA units in PHG structure help to maintain the network hydrophilicity. Figure S3 shows the swollen-state images of hybrid PHG/Mmt hydrogels after their swelling in water. The swelling ratio of the hybrid gels prepared at −18 °C under low-temperature conditions was generally higher than that of hydrogels. The hybrid gels first tended to swell with increasing Mmt content up to 1.10% (*w*/*v*) and then decreased monotonically. The observed decrease is due to the nanoclay particles acting as physical crosslinking points within the PHG network, in addition to the chemical crosslinker TEGDMA.

The relatively increased crosslinking further reduces the free volume within the hybrid structure, making it more difficult for water molecules to enter, resulting in lower swelling rates. It is widely known that increasing the Mmt weight ratio up to 4% increases the water absorbency, while increasing it further decreases the absorbency. A similar decreasing trend was reported for PGMA/Na–montmorillonite nanocomposites prepared by changing the GMA content [37]. The observed swelling reduction showed a monotonic change depending on the clay content added to the hybrid structure. As the polymer amount entering the layers reached a limit, the clay interlayers opened to their maximum, resulting in a crosslinked structure that prevents the water molecules from entering the structure.

**Figure 6:**
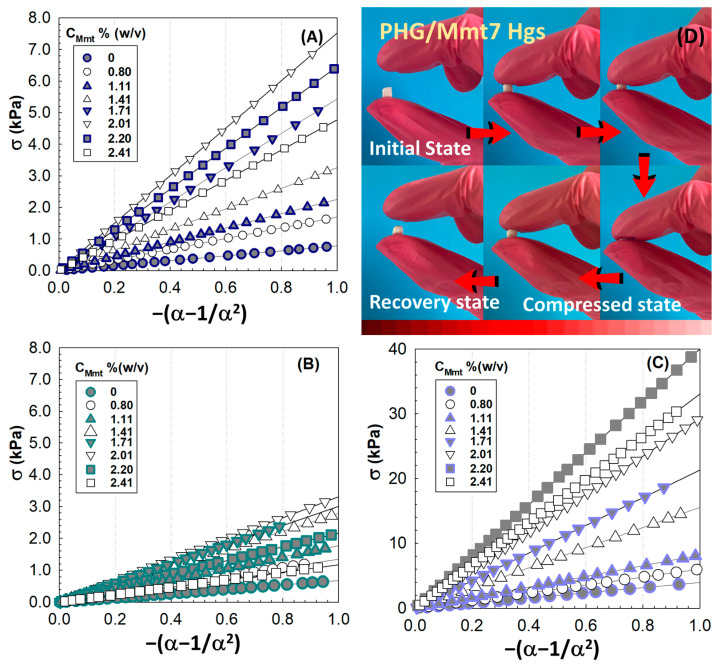
Isothermal compression curves of compressive stress as a function of the hybrid composition as-prepared state measurements of hydrogels (**A**), and swollen-state measurements of hybrid hydrogels (**B**) and that of hybrid cryogels (**C**). Optical images of 2.41% (*w*/*v*) Mmt-loaded hybrid hydrogels during manual compression (**D**).

**Figure 7:**
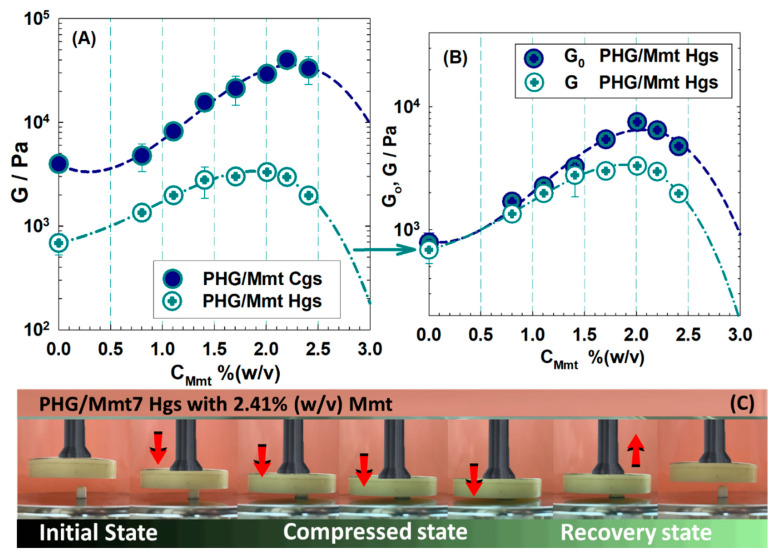
Elastic modulus of Mmt-loaded hybrid hydrogels and cryogels after their swelling in water (**A**), and comparison of as-prepared state and swollen-state modulus of hybrid hydrogels (**B**). The values are mean ± SD, *n* = 4. Error bars are not visible if they are the same size or smaller than the symbols. (**C**) Optical images of 2.41% (*w*/*v*) Mmt-loaded hybrid hydrogels during uniaxial compression.

To evaluate the effect of Mmt integration into the PHG matrix, the uniaxial compression testing was performed at 0.5 mm/min and room temperature after preparation stage and after their equilibrium swelling in water. Figure 6 presents the isothermal compression curves of compressive stress as a function of the hybrid composition. Compared to the as-prepared state condition (Figure 6A), the compressive stress required to compress the swollen PHG/Mmt hydrogels to 10% of their original size decreases (Figure 6B) and also varies with the amount of inorganic nanolayers of Mmt added to the PHG structure. However, to compress the hybrid PHG/Mmt cryogels synthesized under cryoconditions to the same extent, greater compression force is required (Figure 6C). Figure S4 presents the optical images of blank PHG Hgs and Cgs during finger compression. The cryogels with sponge-like macroporous structure were typically synthesized by cooling the pre-gel solution at temperatures below the freezing point of the polymerization solvent. A significant portion of the pre-gel solution is concentrated, called cryoconcentration, in unfrozen liquid microphase, while a large amount of the solvent crystallizes and forms crystallized macro-phase. The cryoconcentration of pre-gel mixture makes cryogelling more efficient by reducing the critical concentration needed for cryogelation, and by this way, accelerating the gelation. The pre-gel mixture of higher polymer concentration increases the stiffness of cryogels and also results in the formation of a smaller average pore size. Upon crosslinking, the solvent crystals melt at room temperature, and this thawing process results in an interconnected macroporous structure formation. The pore size and interconnectivity mainly depend on the cryogelation conditions, the type and content of the monomers, the initial solvent crystallization temperature, the freezing point of polymerization solvent, the cryoconcentration of constituents in pre-gel, the extent and rate of crosslinking, and the temperature and duration of the cryogelation process [38,39,40,41]. The average gel strength was assessed by the compressive modulus after preparation *G_0_* and after the swelling-state obtained from Equation (3). The results, shown in Figure 7, indicate that the *G* values increase with the increase in Mmt concentration up to 2.20% (*w*/*v*). Then, at values above this content, the gel strength decreases, that is, the hybrids would become weaker when the Mmt content exceeds 2.20% (*w*/*v*). This is because excessive Mmt content due to agglomeration can cause the hybrid matrix to become discontinuous. The incorporation of 2.20% (*w*/*v*) Mmt resulted in a 10-fold increase in the elastic modulus of hybrid cryogels from 0.39 kPa to 40.1 kPa, while it resulted in a 4.3-fold increase in the modulus of hybrid hydrogels. The hybrid PHG/Mmt structure primarily consists of copolymeric PHG chains that interconnect adjacent Mmt layers, and the copolymeric chains are effectively crosslinked by the Mmt layers. In the blank PHG gel structure, the network chains are randomly crosslinked only by TEGDMA units, resulting in a wide distribution of length of the network chains between random crosslinking points. In contrast, the flexible polymer chains within the structure exhibit random conformations depending on the distance between the Mmt layers.

The chain lengths between the Mmt layers change proportionally to the distance between the clay particles, resulting in the unique polymer/clay network structure and exceptional mechanical properties. Similar results were reported in the study where the reinforcing properties of highly exfoliated Mmt/poly(glycidyl methacrylate) nanocomposites were determined, and dynamic mechanical measurements of reinforced epoxy matrix revealed that the addition of low amounts of nanofiller from 1% to 5% wt led to significant improvements in the mechanical properties and an increase in the storage modulus [15]. In similar systems, the deterioration in the mechanical properties with increasing clay content was attributed to the formation of some particles formed as a result of the agglomeration of nanofillers. It was observed that the formation of larger particles was more pronounced in the composites containing 5% wt filler, weakening the mechanical properties. Nikolaidis and coworkers investigated the mechanical properties of organomodified montmorillonite (OMmt)/poly(methyl methacrylate) (PMMA) nanocomposites prepared by in situ bulk polymerization [42]. Evaluation of the tensile measurements indicated an increase in the Young’s modulus and a decrease in the tensile strength and elongation at break, probably due to the formation of hydrogen bonds between the hydroxyl groups in clay and carbonyl groups in PMMA. Although the addition of clay increased the hardness of the hybrid system, the interface adhesion between PMMA and OMmt was not strong enough to withstand large deformations, leading to the premature failure of the nanocomposites and decreased the tensile strength.

### 2.5. Responsive Swelling Properties of Epoxy-Functional Hybrid Gels

**Figure 8:**
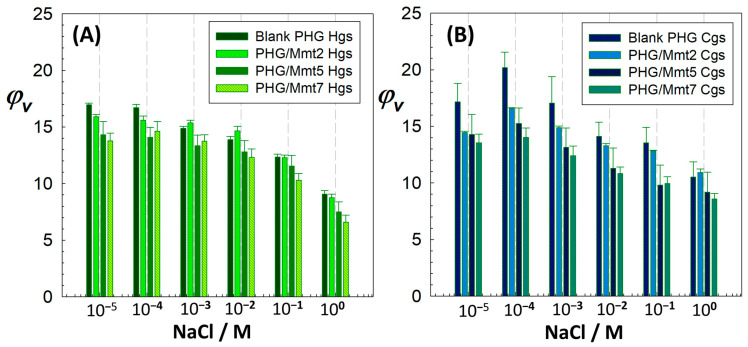
Salt-induced swelling of hybrid PHG/Mmt Hgs (**A**) and Cgs (**B**) with different composition in aqueous NaCl solutions. The values are mean ± SD, *n* = 4.

Swelling properties of the prepared hybrids were examined in aqueous salt (NaCl) and surfactant (CTAC, SDS, and CTAB) solutions at different concentrations. While increasing salt concentration decreased the extent of swelling, increasing the Mmt content showed the same effect. Figure 8 shows how the effect of salt concentration in the external solution on the swelling varies with the composition. The hybrid gels tended to reduce their volume by “salting-out” type swelling at all salt concentrations. For 2.41% (*w*/*v*) Mmt-doped PHG/Mmt7 hydrogel, the volumetric swelling decreased 2.1-fold from 13.780 ± 0.682 to 6.521 ± 0.585 by increasing the concentration of NaCl solution from 10^−5^ M to 1.0 M. For the PHG/Mmt7 cryogel sample, the swelling was reduced by 1.5 times in the same range from 13.557 ± 0.747 to 8.593 ± 0.490. The comparison of shrinkage tendencies in 1.0 M salt solution showed that the hybrid cryogels have less tendency to collapse compared to the hybrid hydrogels due to their porous structure. In similar systems investigating the salt sensitivity of sulfonated polyacrylamide/sodium montmorillonite hydrogels containing different clay concentrations, it was reported that the nanocomposites showed lower salt sensitivity compared to unfilled gels [43]. The reduction, which was more pronounced in the clay-free gels, was due to higher mobility of the network chains, thus exhibiting a higher deswelling behavior. Based on the swelling thermodynamics, different components of the free energy of hydrogels (mixing, elastic, ionic, and Coulomb free energies) vary with the salt concentration in the swelling medium. In the region of low salt concentrations, the elastic free energy of gels varies rapidly with the salt concentration, due to the strong coupling between the chemical and mechanical energy. However, in the region of high salt concentrations, it does not change significantly because of the weaker chemical–mechanical coupling as the network collapses. In their study, Zhang and colleagues experimentally investigated the potential of hydrogels to generate energy from salinity gradients, supported by a theoretical framework [44]. By applying the mechanical pressure to delay the swelling or deswelling of hydrogels in saline solutions, a perspective on the conversion of free energy mixing into the mechanical work is presented. Under external pressure, the mechanical work per unit change in salt concentration is high for dilute solutions and decreases at high salt concentrations, where the effect of salt concentration on the hydrogel volume is minimal.

**Figure 9:**
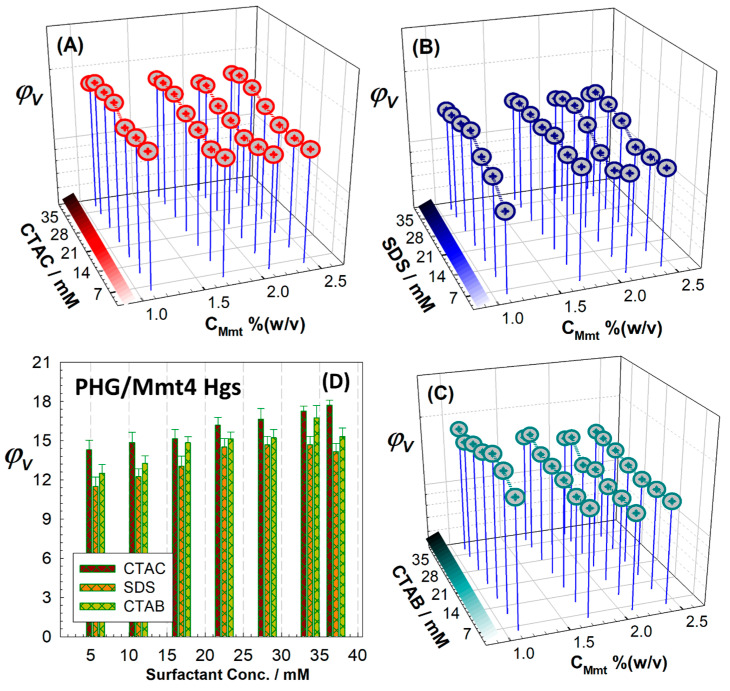
The equilibrium volume swelling ratio of hybrid hydrogels with the surfactant CTAC (**A**), SDS (**B**), and CTAB (**C**) solutions at different concentrations, and comparison of swelling of 1.71% (*w*/*v*) Mmt-doped PHG/Mmt4 Hgs in surfactant solutions (**D**). The values are mean ± SD, for *n* = 3. Error bars are not visible if they are the same size or smaller than the symbols.

Surfactant-regulated swelling of hybrid PHG/Mmt gels was investigated in three different ionic surfactants: anionic SDS and cationic CTAC and CTAB with various concentrations. Figure 9 compares the equilibrium swelling ratio of hybrid hydrogels depending on the type of surfactant, while the results of hybrid cryogels are presented in Figure S5. The swelling pattern of the hybrids indicates that these gels exhibit good swelling performance with both anionic and cationic surfactants, with good stability. Considering the nature of the surfactants, the hybrid gels tend to swell more in cationic CTAB and CTAC solutions than in anionic SDS, with the highest swelling observed in CTAC solutions. Addition of inorganic Mmt to the PHG matrix reduced the swelling, regardless of the nature of the surfactants. In surfactant medium, the swelling capacity increased 1.2 times as a result of salting-in behavior in all surfactant solutions by changing the surfactant concentration between 5.625 mM and 37.125 mM. Nonionic gels exhibit properties similar to polyelectrolyte gels, as bound ionic surfactants at the gel interfaces create electrostatic interactions. The surfactants bound to the hydrophobic units of the network transform into hydrophilic units that interact specifically with adjacent water molecules, making the network more extensive. Lee and Bae proposed a thermodynamic framework for the swelling behavior of hydrogels in the ionic surfactants SDS and dodecyltrimethylammonium bromide, consisting of the specific interaction and interfacial contribution of the bound surfactant, which explains the effects of surfactant binding on the degree of swelling [45]. The binding of hydrophobic tails from surfactants into the network structure results in the formation of aggregates that alter the interaction capabilities and interfacial properties. From the interface perspective, the monolayers formed by the ionic head groups of the bound surfactants contribute to the electrostatic and interfacial properties [46]. From the polymer chain perspective, the substitution of hydrophilic units from surfactant aggregates significantly disrupts the interaction between the water and polymer gel. Due to the limited occupancy of binding sites by added surfactant molecules, the interaction ability depends on the proportion of occupied binding sites [47].

### 2.6. Adsorption Properties of Epoxy-Functional Hybrid Gels

**Figure 10:**
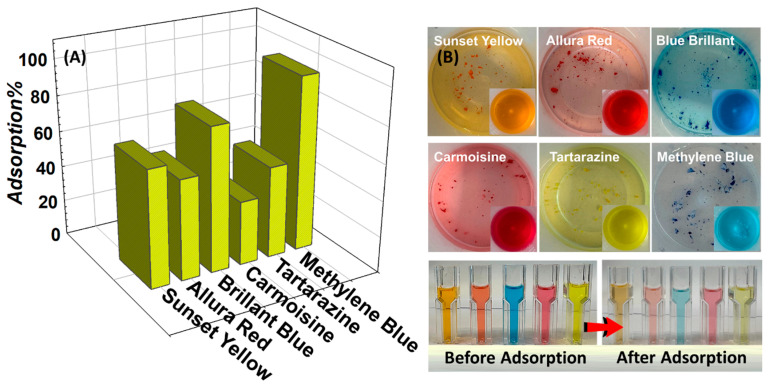
(**A**) Comparison of adsorption% of several azo dyes onto hybrid PHG/Mmt5 Cgs, (**B**) optical views of hybrid gels after reaching the equilibrium state. (Fixed experimental conditions: dye concentration: 20 mg/L, adsorbent mass: 20 mg, volume of solution: 10 mL, contact time: 24 h, stirring speed: 180 rpm and T: 22.8 °C).

After the polymerization was completed, the small cylindrical samples were prepared from rod-shaped gels with a length of 60 mm and a width of 4.0–4.5 mm extracted from the polypropylene syringes and subjected to the swelling and elasticity tests; the samples were then dried, crushed, and turned into powder to be used in the adsorption experiments. The prepared hybrids were tested for their capacity to remove synthetic azo food dyes; tartrazine (E102), brilliant blue (E133), allura red (E129), carmoisine (E122), and sunset yellow (E110), which are popular as food colorants due to their low cost and stability, were compared with the cationic azo dye MB in Figure 10. While the food dyes are important to the food industry primarily due to their coloring properties related to their chemical structure, azo dyes play a key role. From an environmental perspective, the dye waste degrades the water quality, causing serious health problems, particularly for humans, plants, animals, and aquatic life. The environmental importance of using food dyes is that their waste affects the aquatic ecosystems and contaminates drinking freshwater. Various in vivo and in vitro studies, based on acceptable daily intake data and food dye toxicology research, have also demonstrated dye toxicity [48]. Carcinogenic substances in some food dyes damage cells, leading to a higher risk of health problems such as cancer, and can increase hyperactivity in children and cause allergic reactions. While it is possible to replace synthetic dyes with natural colorants, the technological and economic advantages of azo dyes may outweigh the advantages of natural dyes when commercialized as colorants for the food industry [49]. Therefore, the effectiveness of the prepared (alkylmethacrylate)-based hybrids as alternative adsorbents in removing cationic and anionic dyes was evaluated and the adsorption of these dyes by the same adsorbent was compared. It is believed that this will enable the understanding and comparison of the dye-classified adsorption process for a fixed system. In comparison to anionic dye removal of different food dyes in water at 20 mg/L concentration, the highest adsorption was observed in brilliant blue (80.4%) and the lowest adsorption was observed in carmoisine (35.8%). Although the efficiency for other food dyes was sunset yellow (65.4%), allura red (56.2%), and tartrazine (51.2%), the adsorption kinetic studies were continued with MB dye based on the effectiveness of cationic MB dye being 92.3%. The permanent negative charges on the surface of Mmt platelets and the pH-induced charges on OH sites at the fracture edges cause charge heterogeneity, leading to different activities towards dyes. In their study comparing the adsorption of sulforhodamine G, rhodamine B, orange II, and MB onto Mmt, Fang and coworkers reported that the Al^3+^ polarization of the edge hydroxyls caused a water molecule to bridge a tertiary amine group on RhB to a hydroxyl group on the edge surfaces of Mmt. The importance of high polarity edge hydroxyl in the complex formation, hydrogen bond formation and its effects on the adsorption of nitrogenous dyes, and the role of edge surfaces in the adsorption process are stated in [50].

**Figure 11:**
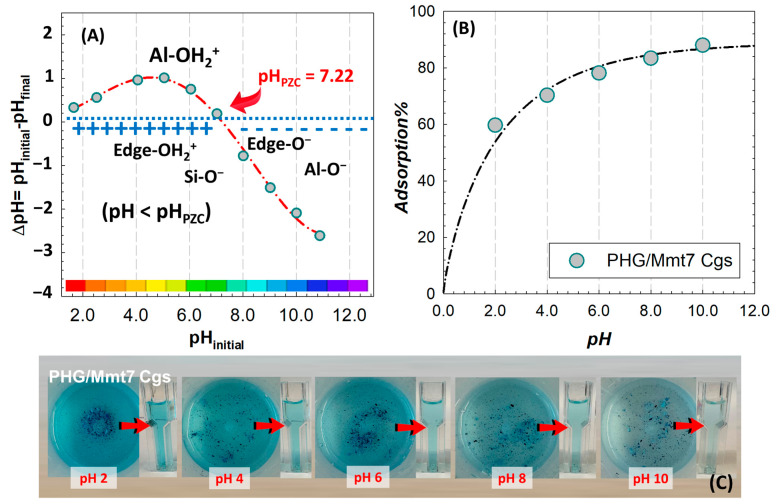
(**A**) PZC point determination plot of ∆pH vs. initial pH for hybrid PHG/Mmt7 Cgs, (**B**) effect of pH on the adsorption properties. (**C**) Optical views of hybrid cryogel samples after reaching the equilibrium state at different pHs. (Fixed experimental conditions: dye concentration: 10 mg/L, adsorbent mass: 20 mg, volume of solution: 10 mL, contact time: 24 h, stirring speed: 180 rpm and T: 22.8 °C).

For the evaluation of the point of zero charge (PZC) of the prepared hybrid structures by characterizing pH-dependent charge change in edge regions in the present Mmt-doped hybrid system, the variation in ∆pH with respect to the initial pH is given in Figure 11A. The Mmt layers have permanent negative charges in the basal planes due to the isomorphic substitutions, which cause the development of charges in the octahedral Al–OH and tetrahedral Si–OH groups at the edges depending on pH variation. These amphoteric sites are conditionally charged, supporting adsorption by developing variable positive or negative charges at the edges through direct H^+^ or OH transfer from the aqueous phase, depending on pH. As seen in Figure 11A, the edge OH groups have a PZC of 7.22 for PHG/Mmt7 Cgs less acidic than the Si–OH groups and less basic than the Al–OH. At pH < 7.22, the surface of the PHG/Mmt matrix becomes positively charged in a protonation reaction of Al–OH sites at edges through the reaction Si-OH + OH- → SiOH_2_^+^ and Al-OH + H^+^ ⇔ Al-OH_2_^+^ and suitable for the adsorption of anionic dyes such as sunset yellow, allura red, brilliant blue, carmoisine, and tartrazine. At higher pH values by deprotonation of Si–OH than that of the Al–OH sites, the negative charging at edges through the reactions Si-OH + OH^−^ ⇔ Si-O^−^ and Al-OH + OH^−^ ⇔ Al-O^−^ increases the activity towards cationic dyes such as MB. Tombacz and Szekeres modeled the charge evolution at the edges and interpreted the pH-dependent surface charge heterogeneity. By analyzing the specific effect of pH on the surface charge of Mmt dispersed in different electrolyte solutions, the zero charge point of edge regions (pH_PZC_, edge) was determined as pH 6.5 [51]. Figure 11 illustrates the way solution pH affects the cationic MB dye adsorption of Mmt-doped hybrid gels. pH has a significant impact on the adsorption process, with the dye removal efficiency of hybrid gels increasing from 59.7% to 87.9% as pH increases. The increase in the number of negatively charged adsorbent sites is due to the increasing deprotonation of the silanol groups on the surface as the pH of the adsorption system increases. The presence of excess H^+^ ions competing with dye cations for the adsorption sites is reflected in the decrease in MB adsorption at acidic pH. The interaction with the cationic dye decreases due to the higher amount of positive charge at the edges of the Mmt lamellae as the pH decreases. Figure 11C presents the color change in the MB solutions in different pHs. As the pH decreases, adsorption efficiency weakens due to the attraction between the positively charged edges and the negatively charged basal plates, which becomes stronger. The relatively high removal rate observed in pH 2 medium (below 60%) is due to the surface hydrogen bond formation between the nitrogen atoms on methylene blue and hydroxyl group on the clay surface. For the present hybrid PHG/Mmt gels, the maximum uptake of MB occurs around pH 10; therefore, the subsequent adsorption kinetics and isotherm tests were carried out at this pH condition. Almedia and coworkers also obtained similar results for the removal of MB from colored wastewater by adsorption onto the Mmt clay; the adsorbed percentage of MB increased with pH and was maximum at pH 11 [52].

### 2.7. Adsorption Kinetics of Epoxy-Functional Hybrid Gels

**Figure 12:**
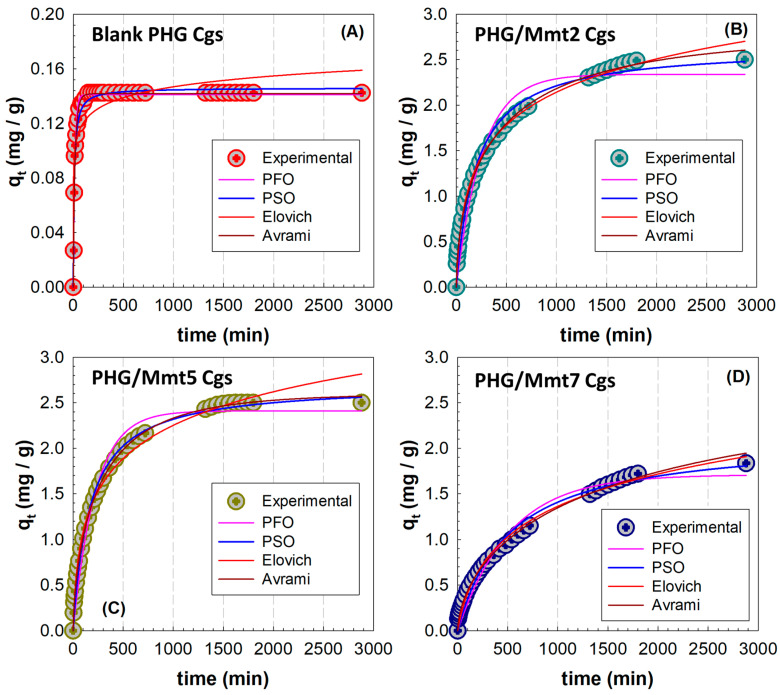
Nonlinear PFO model, PSO model, Elovich kinetic model, and Avrami kinetic model of blank PHG Cgs (**A**) and Mmt-doped hybrid PHG/Mmt Cgs with Mmt concentrations of 1.11% (**B**), 2.01% (**C**), and 2.41% (*w*/*v*) (**D**).

**Figure 13:**
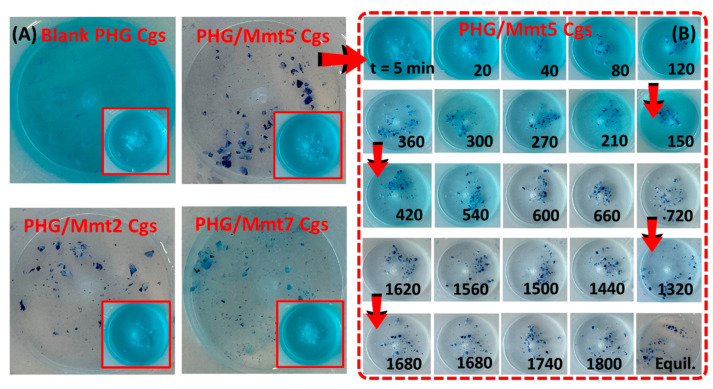
(**A**) Optical views of blank and Mmt-doped hybrid cryogels at equilibrium state and (**B**) time-dependent adsorption of 2.01% Mmt-doped PHG/Mmt5 cryogels during adsorption. (Fixed experimental conditions: dye concentration: 10 mg/L, volume of solution: 10 mL, adsorbent mass: 10 mg, contact time: 48 h, pH: 10, stirring speed: 140 rpm and T: 22.5 °C).

Adsorption kinetics was studied using experimental models to determine the reactions and extent of interactions occurring between the adsorbate and adsorbent. Adsorption kinetics were performed using 10.00 mg (±0.06) of blank PHG gels and hybrid PHG/Mmt Cgs with Mmt doping at 1.11%, 2.01%, and 2.41% (*w*/*v*) Mmt concentrations. The mass optimization was performed by ensuring that these gels were added to an initial concentration of 10 mg/L for cationic MB solutions with a solution volume of 10 mL at pH 10 and 22.5 °C. The experimental data for MB removal by blank PHG (Figure 12A), PHG/Mmt2 (Figure 12B), PHG/mmt5 (Figure 12C), and PHG/Mmt7 (Figure 12D) were fitted to the nonlinear kinetic models of Pseudo-first order (PFO), Pseudo-second order (PSO), and the Avrami and Elovich kinetic models (Table 3). To determine the mechanistic behavior of the process, the experimental data were examined by a regression of intraparticle diffusion onto a multiple linear model, as shown in Figure S8. These kinetic models are mathematically represented as Equations (S1)–(S5) in Table S2. Adding hybrid gel to the MB solution gradually increased the adsorption efficiency as the exposure time increased. The equilibrium was reached after 24 h, and the results revealed that the integration of Mmt enhanced the dye removal efficiency of the PHG structure. Figure 13 presents the color change in the solutions in time. Figure S7 also presents the optical views of time-dependent adsorption of blank and hybrid PHG/Mmt cryogels containing various amounts of Mmt in the feed. The most effective removal was observed in the PHG/Mmt5 hybrid cryogels containing 2.01% (*w*/*v*) Mmt, while the decrease in the adsorption observed with increasing Mmt can be attributed to the saturation effect. The addition of more Mmt to the PHG structure reduces its ability to adsorb additional dye molecules, resulting in a slower adsorption process.

Based on the nonlinear fittings presented in Figure 12, the kinetic parameters for the adsorption of MB onto Mmt-doped hybrid cryogels were collected in Table 3 for the PFO, PSO, Elovich, and Avrami models. The nonlinear regression data, evaluated using the adjusted coefficient of determination (R^2^), showed good fit for all models examined except PFO. In this model implemented using Equation (S1), the PFO constant *k*_1_ (min^−1^) is related to the adsorption rate and 1/*k*_1_ shows the required time for the process to reach the equilibrium. In the hybrid structure, *k*_1_ increased as the Mmt concentration increased, but the decrease observed in PHG/Mmt7 gel at the highest Mmt content resulted in an increase in the time required to reach the equilibrium, as clearly seen in Figure 12D. However, the best fit was obtained with the Avrami model, which showed the highest *R*^2^ values among the four systems evaluated. Comparing the *R*^2^ values of PFO and PSO models with increasing Mmt, the PSO model gives higher values in all gel systems and the adsorbed amount at equilibrium *q_e_* values match better with the experimental values. The adsorption data of MB removal were well fitted by PSO kinetic given by Equation (S2) in Table S3. The equilibrium rate constant values of pseudo-second adsorption, *k*_2_ (g/mg min^−1^), calculated by this model for PHG/Mmt5 cryogel, showed that the initial rate was 2.18 × 10^−3^ (g/mg min^−1^) and the amount of MB adsorbed at equilibrium was 2.6247 mg/g, which agreed well with the experimental data (2.6036 mg/g) as presented in Table S2. Based on the Avrami kinetic model, Equation (S3) was applied to the experimental data to calculate the Avrami kinetic constant *k_Av_* and compare with the *k*_2_ values calculated from the PSO model. Although both models describe the kinetic mechanisms, the constant *k_Av_* provides a more accurate assessment of mechanism compared to the constant *k*_2_, based on the independence of the Avrami constant from the initial adsorbate concentration (min^−1^). The PSO constant is highly dependent on the initial adsorbate concentration in the solution (g mg^−1^ min^−1^). In addition, the Avrami kinetic model is fractal in type, describing a kinetic system with a time-dependent rate coefficient. Therefore, while the value of *n_Av_* can be integer or fractional, when *n* = 1 the model corresponds to the PFO model. While this value was 0.8350 for the blank gel, the values ranging from 0.5629 to 0.6569 were obtained for the Mmt-doped gels. The adsorption process changes with time depending on the effect of the contribution from multiple stages in the dominant mechanism on the total adsorption rate. Based on the Elovich model, using Equation (S4), the parameters *α* and *β* which are the initial adsorption rate and the desorption constant, respectively, were determined. While the initial adsorption rate increased with the addition of Mmt, a decrease was observed at the highest Mmt addition.

The intraparticle diffusion model was applied using Equation (S5) in Table S2 to explain the adsorption mechanism of MB dye onto Mmt-doped PHG/Mmt cryogels, and the fit results of the experimental data are presented in Figure S8 for comparison. The model indicates a linear adsorption profile encompassing two distinct stages, each represented by a linear equation in Figure S8 for each system. The two stages seen in the plots are attributed to the mass transfer phenomenon underlying MB adsorption: the first stage, with a sharp slope, corresponds to the diffusion of MB molecules into the hybrid PHG/Mmt, while the second stage, with a lower slope, corresponds to the intraparticle diffusion of the dye. In Figure S8, values of *C* ≠ 0 indicate that the diffusion resistance dominates the adsorption process, while higher *C* values indicate a greater influence of the boundary layer on the overall adsorption rate, as in the case of PHG/Mmt5 cryogel. Therefore, the external diffusion appears to be the limiting step in adsorption. The decrease in *C* values with increasing Mmt content, as observed for PHG/Mmt7 Cgs, indicates that the mass transfer process is governed by the intraparticle diffusion and that the adsorption capacity varies proportionally to the square root of time. Application of the experimental data to the intraparticle diffusion model indicated that the adsorption process occurs in two stages, with an increase in the transition time between stages I and II observed with increasing Mmt content. Therefore, while the MB adsorption rate varies between stages, it also suggests that the mechanism controlling the dye diffusion rate dominates each stage. A similar observation was reported recently by Santos and coworkers for MB adsorption onto sodium zeolites. The intraparticle diffusion model significantly influenced the intraparticle diffusion of the boundary layer, highlighting the short transition time between the stages [53].

**Figure 14:**
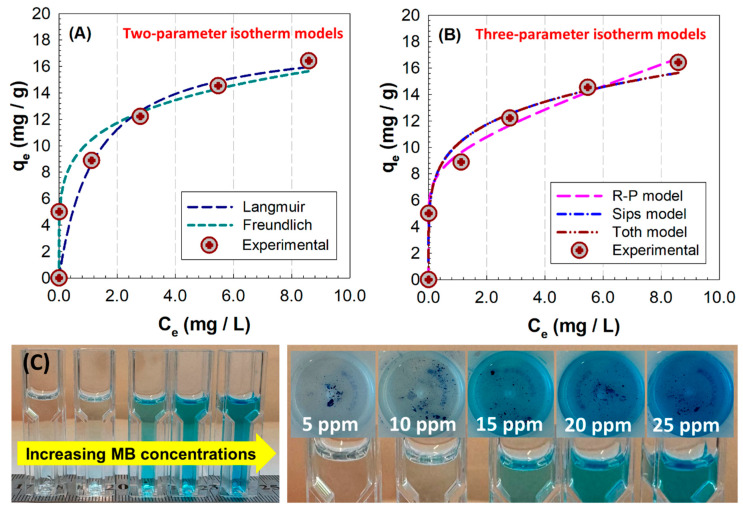
Results of nonlinear two-parameter isotherm models: Langmuir, and Freundlich (**A**); and three-parameter isotherm models: Redlich–Peterson, Sips, and Toth isotherms (**B**) for adsorption of MB to hybrid PHG/Mmt5 Cgs. (**C**) Optical images of hybrid PHG/Mmt5 samples at different MB dye concentrations. (Fixed experimental conditions: dye concentration: 5g–25 mg/L, volume of solution: 10 mL, adsorbent mass: 10 mg, contact time: 24 h, pH: 10, stirring speed: 140 rpm and T: 22.5 °C).

The adsorption equilibrium was analyzed by changing the initial concentration of MB solutions at pH 10 in the range of 5 to 25 mg L^−1^. The equilibrium adsorption capacity of Mmt-doped hybrid PHG/Mmt5 Cgs was calculated using Equation (4). Considering the calculated adsorption capacity and the equilibrium concentration values, the isotherm curves were plotted as *q_e_* versus *C_e_* in Figure 14A and applied to the nonlinear models of Langmuir and Freundlich as two-parameter isotherms, as well as to the Redlich–Peterson, Sips, and Toth model as three-parameter isotherms, using the model equations presented in Table S4. The change in dye adsorption capacity of the hybrid PHG/Mmt5 Cgs sample at initial MB dye concentrations ranging from 5 mg L^−1^ to 25 mg L^−1^ is shown in Figure 14A. The maximum dye adsorption capacity for the hybrid network increased from 5.01 to 16.42 mg g^−1^, while the higher dye removal capacity ranged from 65.7% to 98.9%. The intermolecular forces that developed between the adsorbent and the dye, which facilitated the movement of the dye molecule to the adsorbent surface, increased the removal capacity. This behavior is in agreement with previous results obtained for MB-immobilized poly(glycidyl methacrylate-co-methyl methacrylate) polymer composites. As the MB content increases linearly between 8 and 40 ppm, the polymer composites reach the highest value, i.e., 3.94 mg/g [54]. In addition, considering the negative effect of the increase in the amount of polymer on the amount of immobilized MB, changing the time from 5 min to 30 min had a slight effect by decreasing the amount of MB from 3.65 mg/g to 3.94 mg/g.

Fitting Langmuir model using Equation (S6) in Table S3, the parameters *K_L_* (L/mg) and *R_L_* defined as the Langmuir adsorption constant and Langmuir separation factor, respectively, were determined (Table 4). This model describes the adsorption process as monolayer adsorption on a homogeneous material surface with the same affinity of binding sites. The *K_L_* value was 0.7985, and the calculated *R_L_* value for MB dye adsorbed by hybrid gels was within the range of 0.0437–0.1860, indicating that the adsorption process was easily achieved, as these values were between 0 and 1. However, in the Freundlich model described by Equation (S7), the adsorbent is assumed to have a heterogeneous surface and the adsorption process is considered interactive. Comparing the results obtained for these two models shows that the higher value for the correlation coefficient for hybrid cryogels, *R*^2^ = 0.9734, is obtained in the case of the Freundlich model. This result indicates that the Mmt-doped hybrid cryogel has a heterogeneous surface that allows the interactive adsorption of MB molecules, and it can be concluded that its chemical versatility based on 2.01% (*w*/*v*) Mmt doping leads to adsorbent–adsorbate interactions. In Table 4, the parameter *n_F_* (5.0687) being between 1 and 10 indicates the suitability of the physical adsorption process, while the value of *K_F_* (10.230) indicates the high adsorption capacity of MB by the hybrid cryogel.

The three-parameter models, Redlich–Peterson, Sips, and Toth isotherms, also showed good fit to the experimental data, with *R*^2^ values compared to those given in Table 4. The highest correlation was achieved with the Redlich–Peterson model, while the correlation was lower in the Toth model. Directly comparing two- and three-parameter adsorption isotherms is difficult because each model has different complexity and is formulated based on different hypotheses regarding the adsorption mechanism. Comparisons between the isotherm models with different parameters must be supported by physicochemical interpretation. The Redlich–Peterson isotherm, derived from the Langmuir and Freundlich isotherms, is a versatile model for both homogeneous and heterogeneous systems because it predicts dynamic equilibrium over a wide concentration range. The Redlich–Peterson isotherm approaches ideal conditions of Langmuir isotherm at low concentrations, while it approaches Freundlich isotherm at higher concentrations. Similarly, the Sips isotherm model, a combination of the Langmuir and Freundlich models, addresses the limitations associated with the Freundlich isotherm resulting from increasing adsorbate concentration and is based on describing heterogeneous systems with the heterogeneity factor. Setting the *n_s_* value of 5.0699 in the Sips results implies the multiple adsorption sites/heterogeneous adsorption sites, converging the results to the Freundlich model. In Table 4, the parameters β_RP_ = 0.1135 and *n_S_* = 5.0699 obtained from Redlich–Peterson and Sips models show that the isotherms did not converge to Langmuir because they differed from 1. Therefore, the interactions between cationic MB dye and Mmt-loaded hybrid matrix are best represented by Freundlich isotherm. A similar observation was reported by Tong and coworkers for the adsorption of MB from aqueous solution onto porous cellulose-derived carbon/montmorillonite nanocomposites. Their findings are in better agreement with the Redlich–Peterson model of equilibrium adsorption [55].

### 2.8. Adsorption Mechanism Analysis of Epoxy-Functional Hybrid Gels

**Figure 15:**
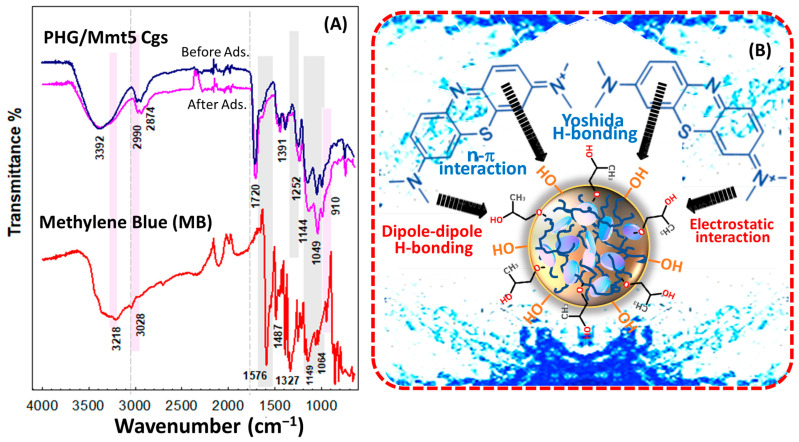
(**A**) FTIR of hybrid PHG/Mmt5 Cgs sample after MB dye removal, where different colors represent the functional groups in the hybrid structures and (**B**) illustration of possible interaction of cationic MB dye and Mmt-integrated hybrid matrix.

FTIR was used to analyze the molecular structure of MB dye and Mmt-integrated hybrid gel before and after MB adsorption (Figure 15). The absorption peak with a maximum at 3218 cm^−1^ was due to the O–H stretching vibration, while the stretching vibration of C–H in methylene was observed at 3028 cm^−1^. The skeletal stretching vibration of benzene ring was observed with a strong absorption peak at 1567 cm^−1^. The characteristic stretching vibration of C–N in aromatic amines was observed at 1487 cm^−1^ and 1393 cm^−1^, while the stretching vibration of C–N in aliphatic chain was detected at 1149 cm^−1^. The split peak with a maximum at 1327 cm^−1^ is attributed to the asymmetric and symmetric stretching vibration of –CH_3_, while the peak at 867 cm^−1^ belongs to the absorption of in-plane bending vibration of C–H [56]. After sorption of the MB dye, the intensity and displacement in the hybrid gel spectra changed slightly, but no new peaks formed, indicating that the adsorbent functional groups remained unchanged. After adsorption, the tensile vibration of C–N in the aliphatic unit near 1149 cm^−1^ both increased and shifted to 1144 cm^−1^. The displacement of the adsorption peaks is observed due to the weakening of stretching and torsional vibrations by the functional groups with increasing volume. The peak intensity at 1389 cm^−1^ belonging to the symmetric bending vibration of CH_3_ groups in the dimethylamine groups increased, while that at 1391 cm^−1^ decreased [57]. Similarly, after adsorption, the intensity of the peak at 1049 cm^−1^ decreased, while the intensity of the peak at 991 cm^−1^ increased significantly, indicating that the absorbance bands of Mmt integrated into the structure around 1049 cm^−1^ and 1006 cm^−1^ are due to vibrations caused by out-of-plane stretching and in-plane stretching of Si-O bonds, respectively. These results suggest that the possible mechanisms for MB adsorption by the Mmt-doped PHG/Mmt gel include the dipole–dipole hydrogen bonding, n-π interaction, and Yoshida hydrogen bonding. In their work, Li and coworkers suggested that hydrogen bonding may not be an important process for MB adsorption in the low-charge Mmt [58]. It has been reported that the parallel orientation of the OH groups may prevent the formation of extensive hydrogen bonds between OH and N when the MB molecules are in a horizontal orientation. Another potential mechanism is the electrostatic interactions between charged groups in Mmt units in hybrid structure and charged groups in MB dye.

### 2.9. Comparison with Other Adsorbents and Their Environmental Impact

Table 5 provides a detailed comparison of other adsorbents including those with resembling structures studied in the literature with the proposed hybrid gel system used in this study for the removal of anionic and cationic dyes. The Mmt-integrated copolymeric PHG structure exhibits effective performance against both anionic and cationic dyes and offers a good adsorption capacity compared to other Mmt-based adsorbents reported in the literature. Various conditions such as the adsorbent dose and surface area, the volume and concentration of the dye solution, and the pH of the environment should be considered in comparison. The future potential of Mmt-containing (alkyl)methacrylate-based adsorbents for dye adsorption is clear, offering significant applications in various sectors. As a naturally occurring clay mineral with a layered structure, Mmt-based hybrid gels exhibit a wide range of adsorptive efficiency for a wide variety of anionic and cationic dyes. Customizing Mmt-based adsorbents by adding new surface layers to their hybrid structure to improve their ability to capture specific dye molecules could lead to the development of highly efficient and selective adsorbents by blending various natural components. Further research in this area could provide significant new insights by further improving the emerging concept.

## 3. Conclusions

A series of epoxy-functional (alkyl)methacrylate-based hybrid gels reinforced with layered silicate montmorillonite were designed by following the free radical copolymerization of GMA, HPMA, and various amounts of Mmt nanoclay in aqueous solution. To provide a perspective on the formation–structure–property relationships, mechanistic investigation was presented as a function of the hybrid composition, and the effect of the amount of nanoclay Mmt added to the reaction system on tailoring the mechanochemical properties of P(HPMA-co-GMA)-based hybrids was investigated. The remarkable improvements in the properties of the hybrids were attributed to the multifunctional network structure based on the tunable mechanical and swelling properties. While the addition of Mmt reduced the degree of swelling of the hybrids in water, the compressive elasticity increased as the Mmt content increased for the hybrids until the Mmt content in the matrix reached 2.20% (*w*/*v*); above this value, the gel strength decreased due to the formation of agglomeration. Mmt-integrated hybrid gels have been shown to have excellent salt-induced swelling response and good salt tolerance in monovalent salt solutions, but further elasticity measurements are needed to determine their mechanistic behavior in salt solutions. Regarding the effect of Mmt concentration and surfactant structure on the swelling ability, in the presence of ionic surfactants, the swelling increased 1.2 times as the surfactant concentration increased, with a “salting out” pattern. While Mmt integration into the structure changes the adsorption efficiency depending on the solution pH, the adsorbed MB increases with pH and reaches a maximum at pH 10. The kinetics of MB cationic dye adsorption followed the PSO model, while the equilibrium adsorption data was found to fit the Freundlich model better. Based on their anionic and cationic dye removal capacity with environmentally friendly structure, the epoxy-functional (alkyl)methacrylate-based hybrid gels designed by integrating inorganic montmorillonite have been proposed as sustainable alternative materials for the removal of different components from wastewater as a solution to water pollution caused by organic dyes, which poses a serious health and environmental threat to the ecosystem.

## 4. Materials and Methods

### 4.1. Materials

Glycidyl methacrylate (GMA, Aldrich, Saint Louis, MO, USA), hydroxypropyl methacrylate (HPMA, Aldrich, Saint Louis, MO, USA), and tetraethyleneglycol dimethacrylate (TEGDMA, Fluka, Darmstad, Germany) were used in the preparation of hybrid gels. Montmorillonite-K10 was supplied by Sigma–Aldrich Co. (St. Louis, MO, USA, CAS Number: 1318-93-0) with a surface area of 220–270 m^2^/g and was used without additional processing. Redox-initiator system consisting of ammonium persulfate (APS, Merck, Darmstadt, Germany) and *N,N,N′,N′*-tetramethylethylenediamine (TEMED, Merck, Darmstadt, Germany) were used to initiate the polymerization. Potassium dihydrogen phosphate (Riedel-de Haen, Seelze, Germany), hydrochloric acid (Merck, Darmstadt, Germany), potassium phosphate (J.T. Baker, Phillipsburg, NJ, USA), and disodium hydrogen phosphate (Merck, Darmstadt, Germany) were used for the preparation of the buffer solutions. Sodium chloride (NaCl, Merck, Darmstadt, Germany) was used for the salt-sensitive swelling experiments; sodium dodecylsulfate (SDS, CH_3_(CH_2_)_11_SO_4_Na, Pittsburgh, PA, USA) as an anionic surfactant and cetyltrimethylammonium chloride (CTAC, [(C_16_H_33_)N(CH_3_)_3_]Cl, Sigma-Aldrich, Taufkirchen, Germany) and cetyltrimethylammonium bromide (CTAB, [(C_16_H_33_)N(CH_3_)_3_]Br, Sigma-Aldrich, Taufkirchen, Germany) as cationic surfactants were used for surfactant-induced swelling studies.

### 4.2. Synthetic Pathway for the Preparation of Epoxy-Functional Hybrid Gels

**Scheme 2:**
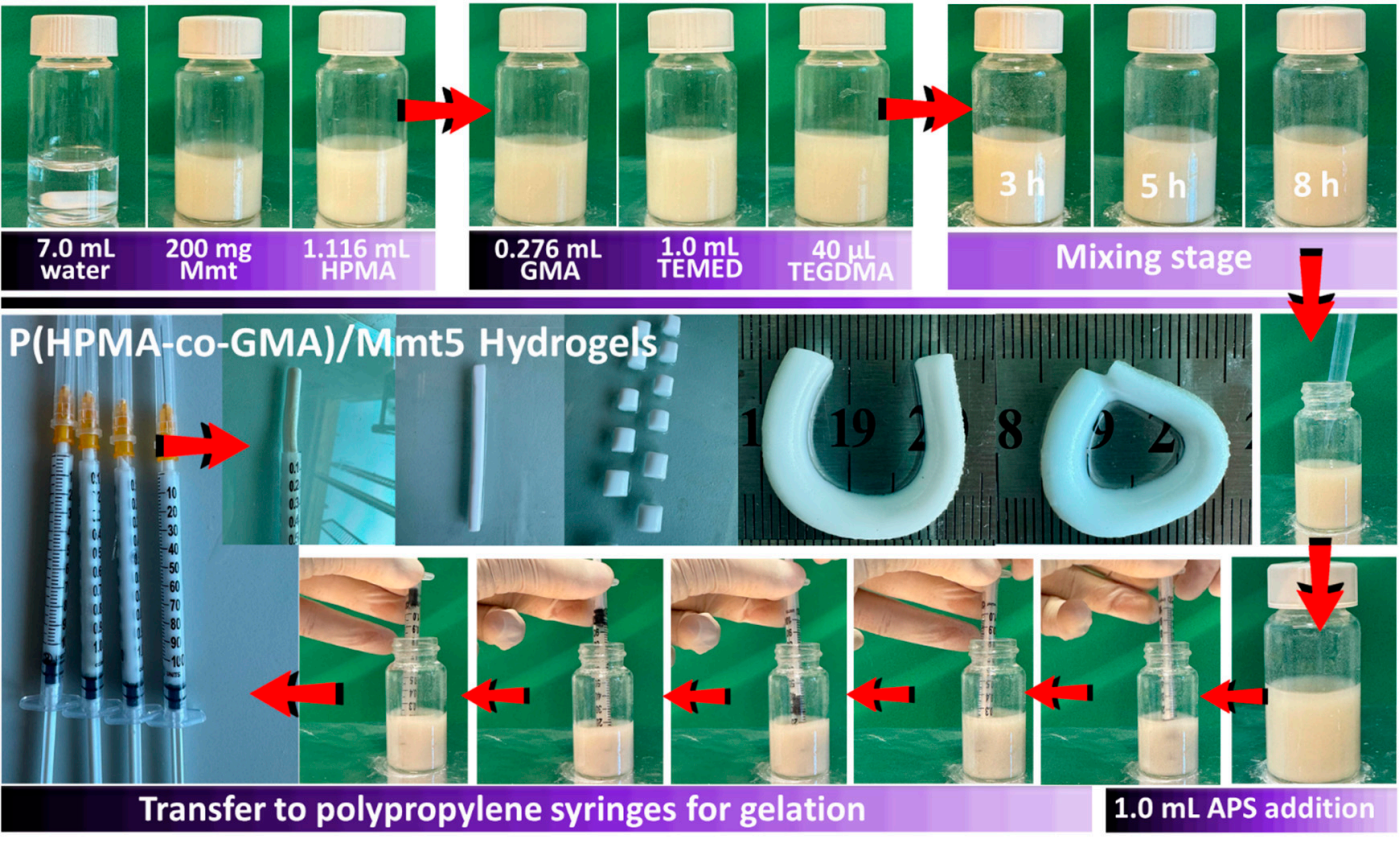
Synthetic pathway followed for the preparation of Mmt-integrated hybrid PHG/Mmt gels, transfer stage to polypropylene syringes for gelation, and as-prepared state images of hybrids after their removal from the syringes.

In this study, the aim was to design hybrid structures with improved mechanical properties based on crosslinked copolymer poly(hydroxypropyl methacrylate-co-glycidyl methacrylate) P(HPMA-co-GMA) using hydrophilic montmorillonite (Mmt) nanoclay as inorganic filler. In the synthesis of hybrid gels, hydroxypropyl methacrylate as the monomer, glycidyl methacrylate as the comonomer, and tetraethyleneglycol dimethacrylate (TEGDMA) as the crosslinker were selected, and hybrid gels were obtained by integrating various amount of pristine Mmt, ranged between 0.80 and 2.41% (*w*/*v*), into the reaction mixture. While the copolymeric network structure was designed with HPMA and GMA molar ratios of 80 and 20 mol%, the crosslinking ratio for the chemical crosslinking of the copolymer, i.e., the ratio of the molar concentration of TEGDMA to the HPMA + GMA concentration, was fixed as 1/78 [21]. Scheme 2 illustrates the synthetic pathway followed for the preparation of Mmt-integrated hybrid PHG/Mmt gels, transfer stage to polypropylene syringes for gelation, and as-prepared state images of hybrids after their removal from the syringes. For the preparation of hybrid PHG/Mmt5 gel, 200 mg of Mmt was first dispersed in 7.0 mL of ultrapure water and stirred for 2 h on a magnetic stirrer. After sequential addition of 8.247 mM HPMA, 2.021 mM GMA, 1.0 mL of TEMED stock solution (24.9 mM), and 0.131 mM TEGDMA crosslinker, the mixture was stirred for another 6 h. During this mixing stage, the appearance of the solution was stable as shown in Scheme 2, and the most homogeneous distribution was achieved. Over a total of 8 h of constant stirring, 1.0 mL of APS stock solution (3.51 mM) was added to initiate the polymerization and then was subjected to rapid stirring for 5 sec. The solution was quickly transferred to polypropylene syringes of 1.0 mL of volume, and the reaction was carried out at 5 °C and at −18 °C for about 48 h to obtain P(HPMA-co-GMA) hydrogels (Hgs) and cryogels (Cgs), respectively.

### 4.3. Swelling Measurements of Epoxy-Functional Hybrid Gels

The resulting cylindrical rods were removed from the syringes as illustrated in Scheme 2 and were cut into small cylindrical samples which were immersed in an excess of water for 3 days to remove the residual impurities and the uncrosslinked polymers. For dehydration, the samples were weighed and allowed to dry until a constant weight was reached. Based on the drying procedure, the characteristics network parameter defined as the volume fraction of crosslinked network after preparation state $\nu_{2}^{0}$ was determined as

(1) $$\nu_{2}^{0}={\left[1+\frac{\left(q_{F}-1\right)\rho }{d_{1}}\right]}^{-1}$$

where *ρ* and d_1_ are the densities of copolymer PHG network, 1.948 g ± 0.045 g/mL, and water, respectively [21]. q_F_ is the dilution degree after gel preparation state defined as the ratio of mass of gel after preparation stage to that of drying-stage. Using dry weights, the gel fraction of hybrids was calculated by w_gel_ (%) = (m_d_/m_i_) × 100. For the determination of $\nu_{2}^{0}$ and gel fraction values, three gel samples were measured for each hybrid concentration, and the average of these values was used. The replicate measurements refer to multiple measurements of the same variable on the same hybrid composition, resulting in correlated values. To determine the equilibrium swelling capacity, the volumetric measurements of gel samples were performed by following the diameter change before and after immersing into water, pH buffer solutions, aqueous NaCl solutions (10^−5^ M–1.0 M), as well as ionic surfactant CTAB, CTAC and SDS solutions (5.625 Mm–37.125 mM). After determining the initial diameter *D*_ini_ with a calibrated digital compass (Mitutoyo Digimatic caliper, 500, resolution: 0.01 mm), the diameter change in the samples immersed in water or solution was followed until the swelling equilibrium. Then, the swollen diameters *D_sw_* at thermodynamic equilibrium were measured, and the equilibrium volume swelling ratio $\varphi_{V}$ was determined by the following equation:

(2) $$\varphi_{V}={\left(D_{sw}/D_{ini}\right)}^{3}$$

Based on this equation, as a defining feature of the hybrid gel system, the swelling capabilities, which are affected by the factors such as the network size, the intercrosslink spacing, and the hydrophilicity/hydrophobicity balance of the structure due to the presence of nanoclays, were determined as a function of the inorganic component Mmt. The mean and standard deviation values were recorded for each measurement.

### 4.4. Mechanical Measurements of Epoxy-Functional Hybrid Gels

To determine how the addition of Mmt nanoclay to the copolymeric PHG structure altered the elastic properties, the uniaxial mechanical measurements were performed both after synthesis and after swelling equilibrium was reached in water. Following a standard experimental protocol, each sample was weighted and diameter/length measured before being placed on a plate and compressed. The applied compressive force, *f*, was defined as *f* = mg, where *m* is the mass of the sample and g is the acceleration of gravity (g = 9.803 m/s^2^). After each compression, the equilibrium compressive stress–strain measurements were simultaneously recorded by the computer. Upon compression, the shortening of the specimen length was measured with a digital comparator (IDC-type Digimatic Indicator 543–262, Mitutoyo), sensitive to displacements of 10^−3^ mm, to determine the difference between the loaded and unloaded lengths. During the compression, the sample was subjected to an initial pressure of 0.01 N to ensure full contact between the circular plate and the sample. The compression tests were then completed with a constant crosshead speed, and based on the extent of compressive force applied to the sample, the nominal stress, σ_nom_, was calculated by

(3) $$\sigma_{nom}=f/S=G(\alpha -\alpha^{-2})$$

where *G* is the compressive elastic modulus, *S* is the cross-sectional area defined as *S* = π(*D*/2)^2^, *α* is the deformation ratio defined as deformed length/initial length of the sample, and the corresponding compressive strain ε is the change in the length relative to the initial length of the gel specimen, i.e., ε = 1 – *α*. All compression measurements were performed at room temperature, 23.5 °C, with a 10 s chain relaxation time after each loading. In these measurements, the samples were compressed to only 20% of their initial length. For test reproducibility, four samples from different syringes were tested for each gel system with varying Mmt concentrations, and the mean and standard deviation values were recorded for each measurement.

### 4.5. Adsorption Efficiency of Epoxy-Functional Hybrid Gels

By performing batch adsorption experiments, the adsorption capacities of hybrid PHG/Mmt gels regarding the removal of anionic and cationic dyes—sunset yellow, allura red, blue brilliant, carmoisine, tartrazine, and methylene blue—were determined by varying different parameters (pH, contact time, and dye concentration). Adsorption kinetics were performed using 10.0 mg of dried adsorbent by placing it in vials containing 10 mL of dye solution at an initial concentration (10–20 mg L^−1^). The vials were gently shaken for 0–48 h at 22.5 °C with a thermostatic shaker at a constant rotation speed of 180 rpm, and the supernatant was collected at predetermined time intervals. Then, the absorbance measurements were carried out with a UV-Vis spectrophotometer (New N4S/N4 UV-Visible Spectrophotometer) to determine the amount of dye remaining in the supernatant. The dye removal efficiency and the amount adsorbed at equilibrium were estimated by

(4) $$Adsorption\;(\%)=\frac{\left(C_{i}-C_{e}\right)}{C_{i}}\times 100\;\mathrm{and}\;q_{e}=\frac{(C_{i}-C_{e})V}{m}$$

where *C_i_,* and *C_e_* (mg L^−1^) represent the concentration of the initial dye solution and at the equilibrium state, respectively. To determine the point of zero charge (pH_PZC_) of the adsorbents, 0.1% HCl and NaOH solution was used to adjust the pH of 0.1 mol/L NaNO_3_ solution. The solutions with pH levels between 2 and 11 were prepared and 20 mg of adsorbent was added. The final pH of the mixtures shaken for 24 h was measured with a pH meter to calculate the ∆pH value. While Pseudo-first-order (PFO), Pseudo-second order (PSO), and Elovich and Avrami kinetic models were used to evaluate the adsorption rates of species onto the adsorbent surface; a more comprehensive description of the primary steps involved in the overall process was presented with the intraparticle diffusion model.

### 4.6. Statistical Analysis

In the synthetic pathway, the eight different gel systems were synthesized at two different polymerization temperatures. The volume change and compression testing of four different samples for each of the 16-gel systems were analyzed, and the mean and standard deviation values were obtained. The data analysis was performed using SigmaPlot11.0 software, and the fit quality of each model was assessed by determining the coefficient of determination (R^2^). The model yielding the highest R^2^ value was considered the most representative of the mechanism.

### 4.7. Structural Analysis of Epoxy-Functional Hybrid Gels

All spectra were recorded on a Perkin Elmer Spectrum 100 FTIR (Perkin-Elmer Inc., Norwalk, CT, USA) spectrometer, using a single reflection ZnSe-ATR. Each sample spectrum was obtained by collecting 64 scans in the range of 600–4000 cm^−1^ at a 4 cm^−1^ spectral resolution. Thermogravimetric analyses were performed using a SEIKO EXSTAR 6200 Model TG/DTA apparatus (Seiko Instruments Inc., Chiba, Japan). The specimens, raw Mmt, blank PHG, and hybrid PHG/Mmt, were heated from 25 to 600 °C at a linear heating rate of 10 °C min^−1^. All the measurements were carried out by placing 8–10 mg of samples in an aluminum crucible in the oven. All runs were performed in a nitrogen atmosphere with a flow rate 150 mL min^−1^. XRD patterns were recorded on a Bruker D8 Advance X-ray diffractometer equipped with Ni-filtered Cu-Kα radiation source (*λ* = 1.7902 Å) operating at 40 kV and 40 mA with a step size of 0.05°. Diffraction patterns were detected with a goniometer speed of 0.5 s per step between 4° and 50° (2θ). pH-dependent measurements were performed with a pH meter (Orion) equipped with a glass-Ag/AgCl combination.

## Figures and Tables

**Table 1:** The structural parameters for hybrid PHG/Mmt hydrogels and cryogels: C_Mmt_ (*w*/*v*%) = the concentration of Mmt in the feed, $\nu_{2}^{0}$ = experimental values of volume fraction of crosslinked polymer network after preparation. The values are mean ± SD, *n* = 3.

		PHG/Mmt Hydrogels		Cryogels
Sample Code	C_Mmt_ (*w*/*v*%)	$\nu_{2,theo}^{0}$	$\nu_{2}^{0}$ ± SD	$\nu_{2}^{0}$ ± SD
Blank PHG	0.0	0.0762	0.0842 (±0.0052)	0.0875 (±0.0128)
PHG/Mmt1	0.80	0.0776	0.0798 (±0.0200)	0.0888 (±0.0198)
PHG/Mmt2	1.11	0.0782	0.0892 (±0.0062)	0.0937 (±0.0093)
PHG/Mmt3	1.41	0.0787	0.0873 (±0.0083)	0.0909 (±0.0123)
PHG/Mmt4	1.71	0.0792	0.0899 (±0.0120)	0.0939 (±0.0058)
PHG/Mmt5	2.01	0.0797	0.0939 (±0.0075)	0.0914 (±0.0224)
PHG/Mmt6	2.20	0.0801	0.0957 (±0.0182)	0.0891 (±0.0108)
PHG/Mmt7	2.41	0.0804	0.0933 (±0.0222)	0.0859 (±0.0152)

**Table 2:** TGA data and decomposition % of clay-free copolymeric PHG and hybrid PHG/Mmt Hgs and Cgs, and the temperatures of maximum values of the rate decomposition (T_max_).

Hydrogels	T_max_ (°C)	Weight Loss (%)	Residual Mass at 800 °C (%)	Cryogels	T_max_ (°C)	Weight Loss (%)	Residual Mass at 800 °C (%)
Raw Mmt	46.4	3.0	85.6				
Blank PHG	342.2	47.3	1.4	Blank PHG	342.1	47.2	2.7
PHG/Mmt7	368.2	46.4	3.6	PHG/Mmt7	325.6	47.9	2.4

**Table 3:** The parameters determined by nonlinear regression treatments of total MB sorption data to PFO, PSO, Avrami kinetic, Elovich model, and intraparticle models.

Pseudo-First Order Model (PFO)				Elovich Model		
Sample	k_1_ × 10^−2^ (min^−1^)		R^2^	α (mg/g min)	β (g/mg)	R^2^
Blank PHG	5.9960		0.9812	4.1767	7.2611	0.7941
PHG/Mmt2	0.3860		0.9485	0.0289	1.8705	0.9949
PHG/Mmt5	0.4470		0.9706	0.0348	1.8582	0.9906
PHG/Mmt7	0.1920		0.9680	0.0061	1.8417	0.9902
Pseudo-Second Order Model (PSO)				Linear Intraparticle Model		
Sample	k_2_ × 10^−1^ (g/mg min^−1^)	R^2^	k_initial_ (mg g^−1^ min^−1/2^)	R^2^	k_later_ (mg g^−1^ min^−1/2^)	R^2^
Blank PHG	6.9749	0.9774	0.0178	0.8252	0.0117	0.2633
PHG/Mmt2	0.0192	0.9800	0.0727	0.9874	0.0105	0.5827
PHG/Mmt5	0.0218	0.9926	0.0833	0.9810	0.0269	0.4299
PHG/Mmt7	0.0092	0.9810	0.0424	0.9989	0.0184	0.8414
Avrami Model				Nonlinear Intraparticle Model		
Sample	$n_{\mathrm{Av}}$	k_Av_ × 10^−1^ (min^−1^)	R^2^	K_diff_ × 10^−1^	*C* (min^−1^)	R^2^
Blank PHG	0.8350	5.8560	0.9843	0.0126	0.1012	0.8159
PHG/Mmt2	0.5629	0.2170	0.9989	0.5141	0.4061	0.9323
PHG/Mmt5	0.6568	0.3490	0.9995	0.5278	0.4618	0.8902
PHG/Mmt7	0.5929	0.0565	0.9971	0.3873	0.0647	0.9885

**Table 4:** Parameters determined through nonlinear regression treatments of experimental data to Langmuir, Freundlich, Redlich–Peterson, Sips, and Toth isotherms for MB adsorption onto clay-doped hybrid PHG/Mmt5 cryogel.

Langmuir model			
q_max_ (mg/g)	K_L_ (L/mg)	R_L_	R^2^
18.27	0.7985	0.1860–0.0437	0.8702
Freundlich model			
K_F_ (mg/g)(mg/L)^−1/n^	n_F_		R^2^
10.230	5.0687		0.9734
Redlich–Peterson model			
*K_RP_* (L/g) × 10^−3^	α_RP_	β_RP_	R^2^
0.6436	8.7797	0.1135	0.9943
Sips model			
q_max_ (mg/g)	*K_S_* (L/g) × 10^−7^	n_S_	R^2^
18.37	0.0480	5.0699	0.9757
Toth model			
*K_T_* (m/g) × 10^−1^	n_T_		R^2^
0.4027	0.8025		0.8025

**Table 5:** Comparison of hybrid PHG/Mmt adsorption efficiency with some other reported adsorbents in the literature.

Adsorbents	Dye Name	Kinetic Model	Isotherm Model	Ads. Dosage	Dye Conc.	q_max_ (mg/g)	Ref.
Poly(methacrylic acid-co-acrylamide)/Cloisite 30B nanocomposite hydrogels	MB	Pseudo-first-order	Freundlich and Langmuir	0.5–4 g/L	10–100 mg L^−1^	32.83	[59]
Poly(acrylic acid-co-2-acrylamido-2-methylpropanesulfonic acid)/Mmt nanocomposite hydrogels	MB	Pseudo-second-order	Redlich– Peterson	20–120 mg/L	10–150 mg L^−1^	215	[60]
Montmorillonite K10 modified by hexadecylamine surfactant	Tartrazine	Pseudo-second-order	Langmuir	0.2 g/L	20–60 mg L^−1^	10.09–31.97	[61]
Fe_3_O_4_ magnetic nanoparticles modified with asparagine	Carmoisine	Pseudo-second-order	Freundlich	0.4–2.2 g/L	40 mg L^−1^	24.1	[62]
Ceria/rice flour/montmorillonite nanocomposites	Brillant blue	Pseudo-second-order	Freundlich	0.2–1.2 g/L	10–60 mg L^−1^	24.69–126.20	[63]
Montmorillonite modified with dimethyldialkyl ammonium	Allura red	Pseudo-second order, Ho and McKay model	Langmuir–Freundlich model	100 mg/L	5 mg L^−1^	4.0	[64]
Plant-mediated (aqueous leaf extract of Azadiratcha indica) Ag-TiO_2_/Na-Bentonite nanocomposites	Sunset yellow	Pseudo-first-order	Langmuir–Hinshelwood isotherm	0.1, 0.2, and 0.3 g/L	10 mg L^−1^ to 50 mg L^−1^	2.9 to 15.2	[65]
Activated carbon derived from cassava sievate biomass	Tartrazine and sunset yellow	Pseudo-second-order	Freundlich	0.1 g/L	150 mg L^−1^	20.83 and 0.091	[66]
Poly(hydroxypropyl methacrylate-co-glycidyl methacrylate)/montmorillonite, P(HPMA-co-GMA)/Mmt gels	Tartrazine, carmoisine, brilliant blue, allura red, sunset yellow, MB	Pseudo-second-order	Freundlich	10–20 mg/L	20 mg L^−1^	10,26, 7.18, 16.09, 11.25, 13.10 and 16.42	This work

## Data Availability

The original contributions presented in this study are included in the article/Supplementary Materials; further inquiries can be directed to the corresponding author.

## Supplementary Materials

The following supporting information can be downloaded at https://www.mdpi.com/article/10.3390/gels11100803/s1, Figure S1: (A) ATR-FTIR spectra of raw Mmt, blank PHG, and hybrid PHG/Mmt7 Hgs containing 2.41% (*w*/*v*) of Mmt; (B) optical images of the hybrid hydrogel sample at the completion of synthesis, removal from the syringe, and preparation of cylindrical samples for applications; Figure S2: Thermograms (TGA) and differential thermogravimetry (DTG) curves of raw Mmt clay (A), clay-free blank PHG Hgs (B), that of hybrid PHG/Mmt7 Hgs (C), and the comparison TGA curves of the thermal decomposition with raw Mmt (C); Figure S3: Swollen-state images of hybrid PHG/Mmt hydrogels after their swelling in water; Figure S4: Optical images of blank PHG Hgs and Cgs during finger compression; Figure S5: The equilibrium volume swelling ratio of hybrid cryogels with the surfactant CTAC (A), SDS (B), and CTAB (C) solutions at different concentrations and comparison of swelling of 1.71% (*w*/*v*) Mmt-doped PHG/Mmt4 Hgs in surfactant solutions (D). The values are mean ± SD, for n = 3. Error bars are not visible if they are the same size or smaller than the symbols; Figure S6: Chemical structures of cationic methylene blue (MB) and anionic sunset yellow, allura red, brilliant blue, carmoizine, and tartrazine dyes, and optical appearances of hybrid gels after reaching adsorption equilibrium; Figure S7: Optical views of time-dependent adsorption of blank and hybrid PHG/Mmt cryogels containing various amount of Mmt in the feed. (Fixed experimental conditions: dye concentration: 10 mg/L, volume of solution: 10 mL, adsorbent mass: 10 mg, contact time: 8 h, pH: 10, stirring speed: 140 rpm and T: 22.5 °C); Figure S8: Adjustment of the intraparticle diffusion model by linear fitting of the experimental data; Table S1: Synthesis protocol and composition of epoxy-functional hybrid gels prepared at various Mmt content; Table S2: The equations used for pseudo-first-order, pseudo-second-order, Elovich, Avrami kinetic, and intra-particle model for total MB adsorption onto hybrid PHG/Mmt gels; Table S3: Thermodynamic parameters for the adsorption of MB dye and adsorption capacity of hybrid PHG/Mmt cryogels calculated from nonlinearized kinetic models; Table S4: Linear and non-linearized forms of the isotherm models applied in the adsorption of MB dye. References [67,68,69,70,71,72,73,74,75,76] are cited in the Supplementary Materials.
