# Supplementary material for: Epoxy-Functional (Alkyl)methacrylate-Based Hybrids Reinforced with Layered Silicate Montmorillonite: From Mechanistic Study to Sustainable Wastewater Treatment
Source: Gels. 2025 Oct 7;11(10):803. doi: 10.3390/gels11100803 (PMC12562478; doi:10.3390/gels11100803)
Supplement: Supplementary file 1 [file gels-11-00803-s001.zip › gels-3889918-supplementary.pdf]

Supplementary Materials

S1. Structural characterization of epoxy-functional hybrid gels

Table S1. Synthesis protocol and composition of epoxy-functional hybrid gels prepared at various Mmt content.

HPMA/GMA mol ratio	80 / 20 mol%
Mmt content	0.80 - 2.41% (w/v)
Crosslinking agent TEGDMA	0.134 mM
Crosslinker ratio X	1/78
APS/TEMED ratio	3.51 mM / 24.9 mM
Polymerization solvent	Water
Polymerization temperature	-18, 5 ^{o}C

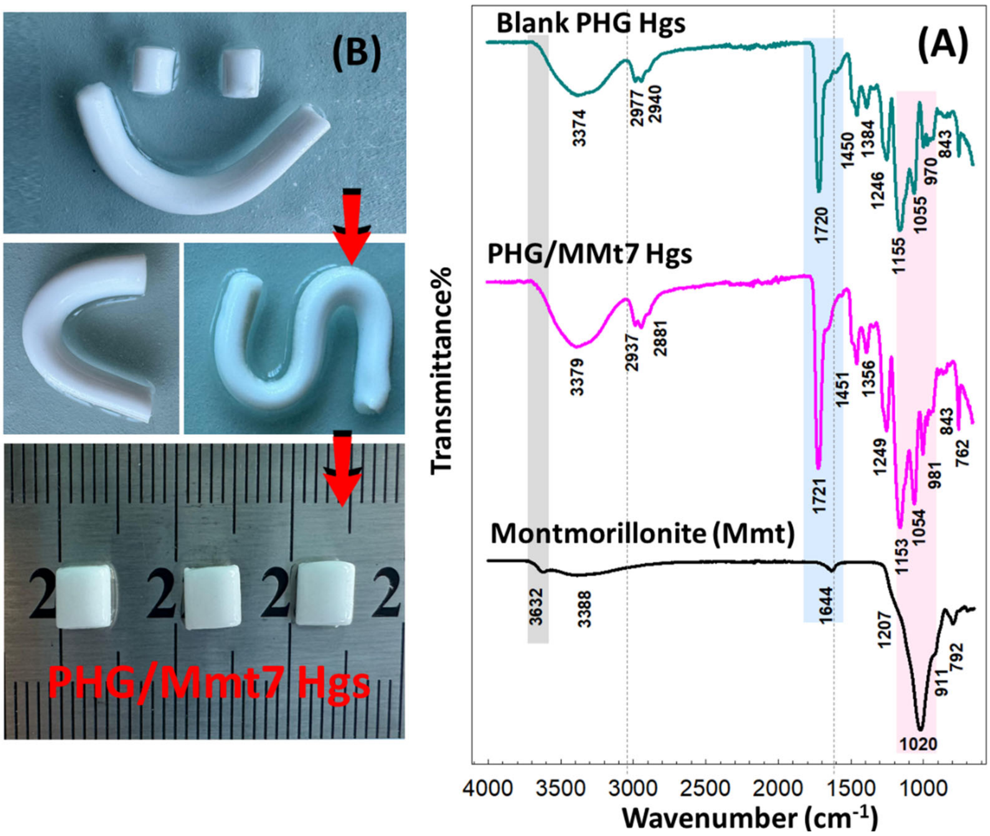

Figure S1. (A) ATR-FTIR spectra of raw Mmt, blank PHG, and hybrid PHG/Mmt7 Hgs containing 2.41% (w/v) of Mmt; (B) optical images of the hybrid hydrogel sample at the completion of synthesis, removal from the syringe, and preparation of cylindrical samples for applications.

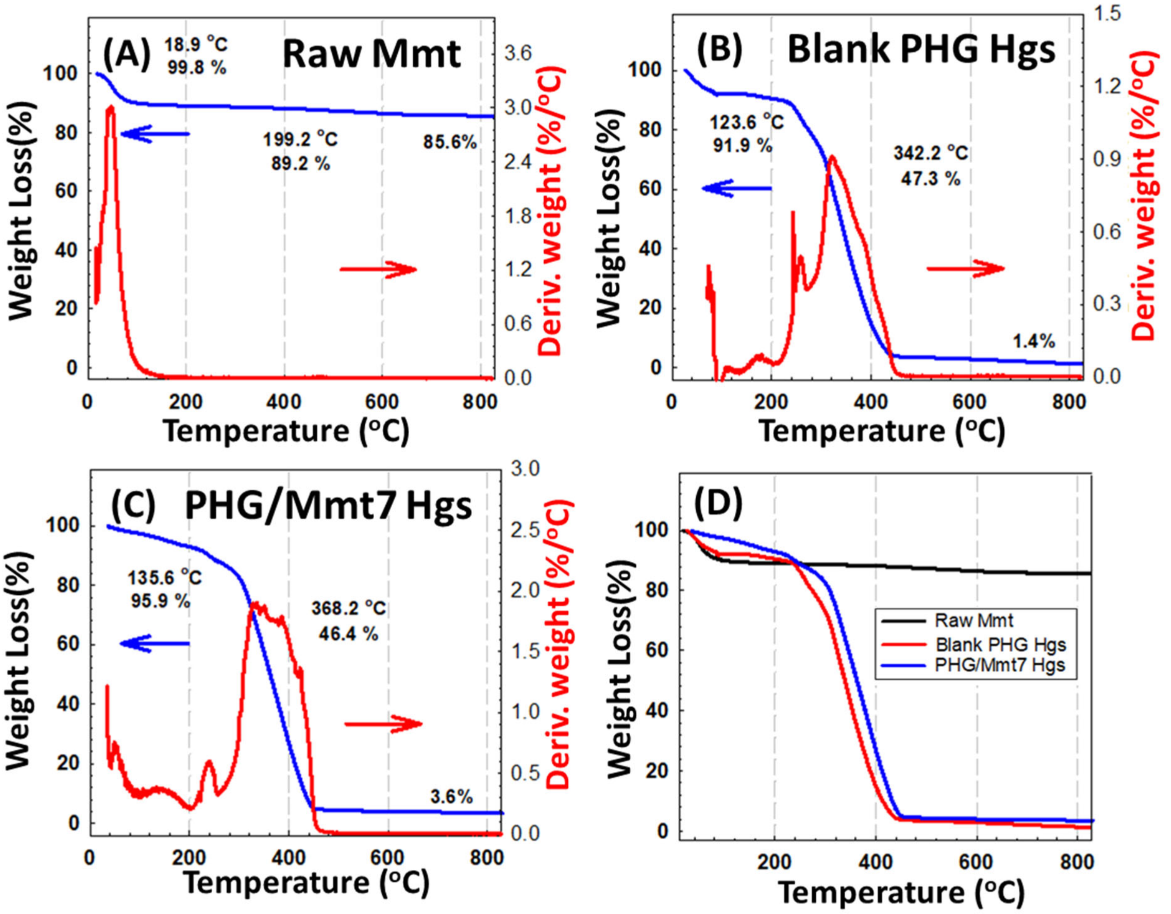

Figure S2. Thermograms (TGA) and differential thermogravimetry (DTG) curves of raw Mmt clay (A), clay-free blank PHG Hgs (B), that of hybrid PHG/Mmt7 Hgs (C), and the comparison TGA curves of the thermal decomposition with raw Mmt (C).

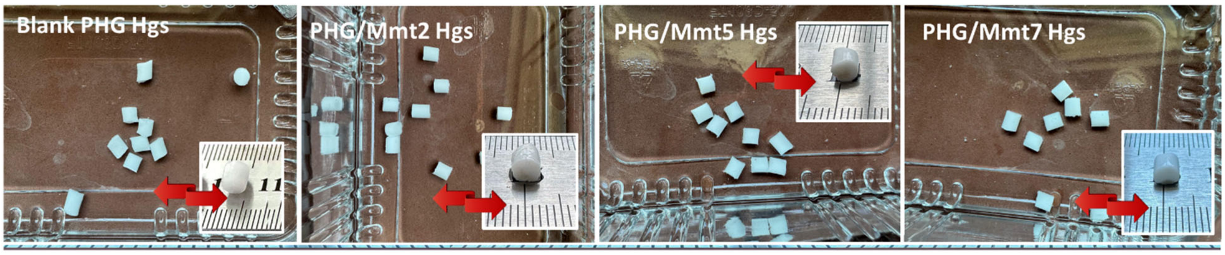

Figure S3. Swollen-state images of hybrid PHG/Mmt hydrogels after their swelling in water.

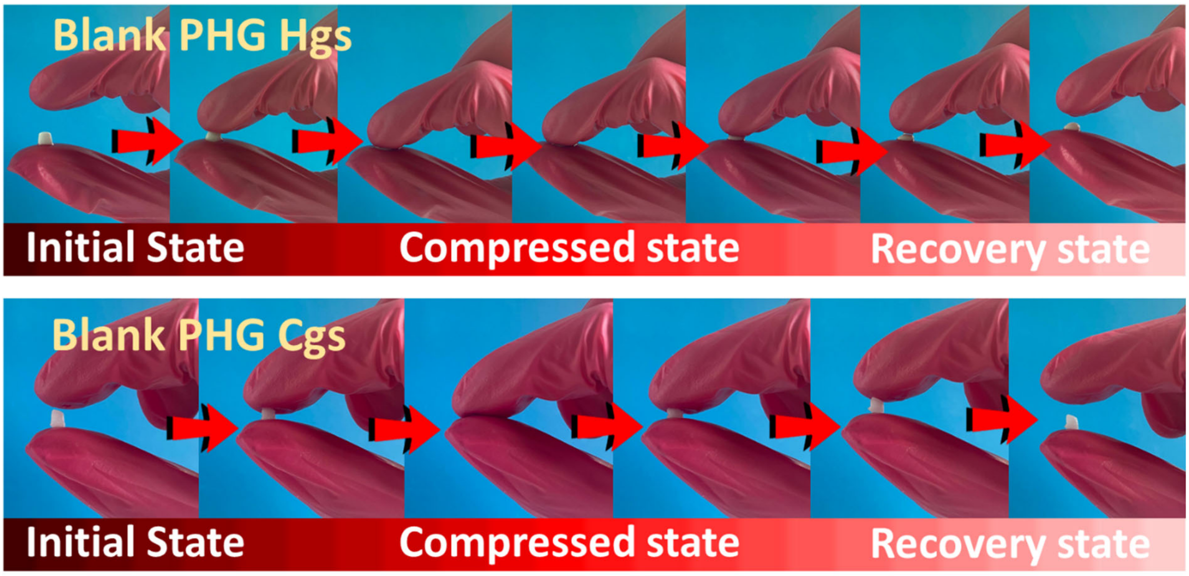

Figure S4. Optical images of blank PHG Hgs and Cgs during finger compression.

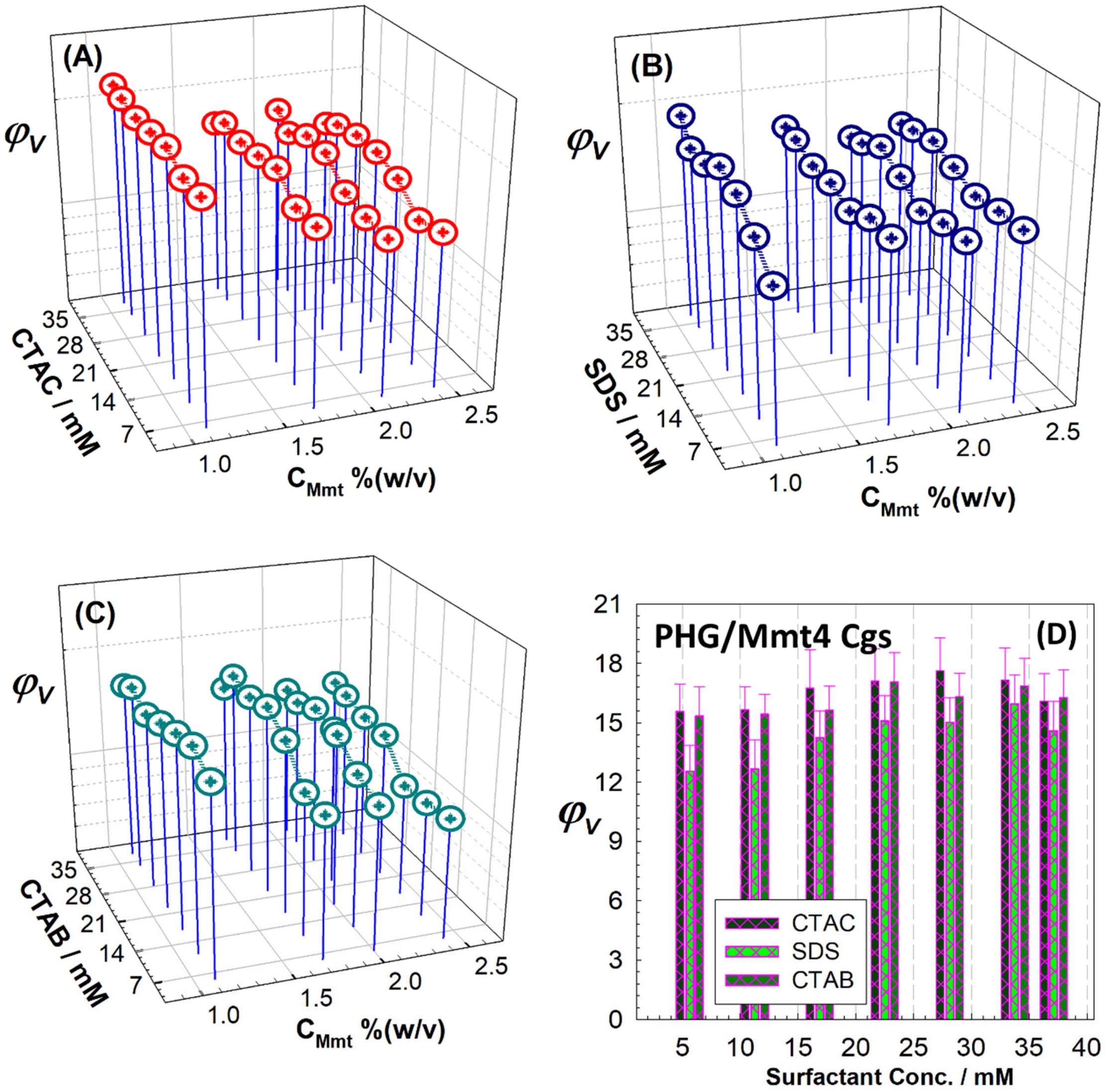

Figure S5. The equilibrium volume swelling ratio of hybrid cryogels with the surfactant CTAC (A), SDS (B), and CTAB (C) solutions at different concentrations and comparison of swelling of 1.71% (w/v) Mmt-doped PHG/Mmt4 Hgs in surfactant solutions (D). The values are mean ± SD, for n = 3. Error bars are not visible if they are the same size or smaller than the symbols

S2. Adsorption properties of epoxy-functional hybrid gels

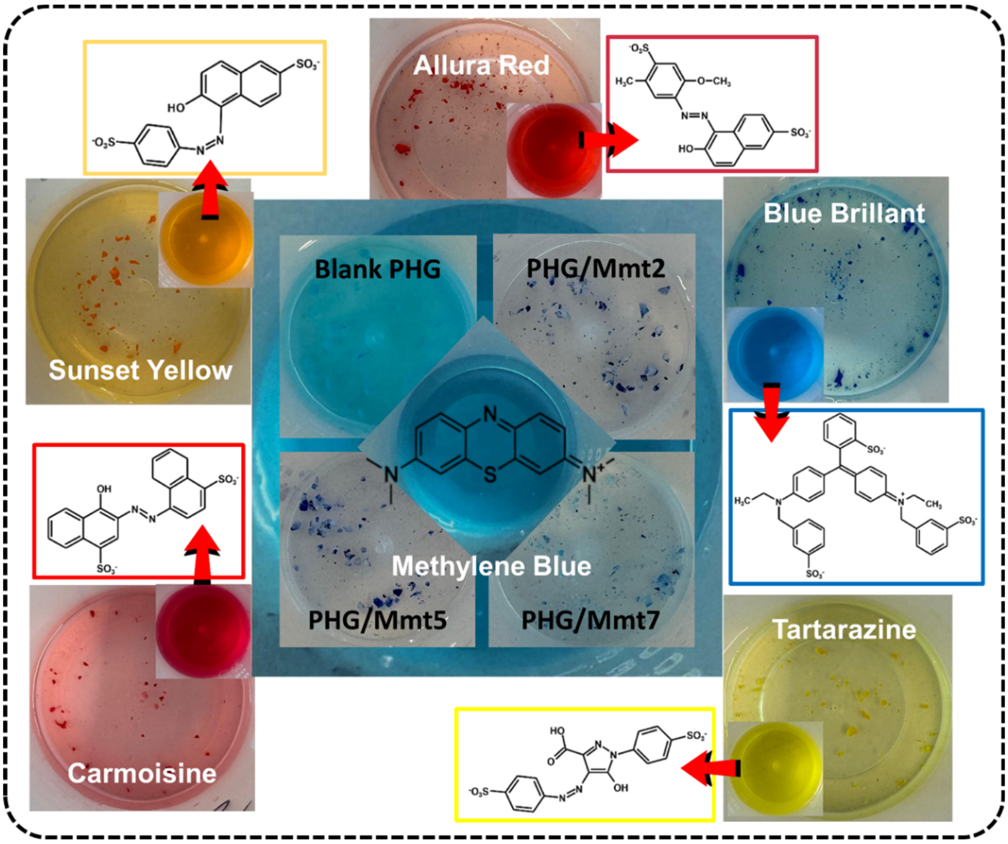

Figure S6. Chemical structures of cationic methylene blue (MB) and anionic sunset yellow, allura red, brilliant blue, carmoizine, and tartrazine dyes, and optical appearances of hybrid gels after reaching adsorption equilibrium.

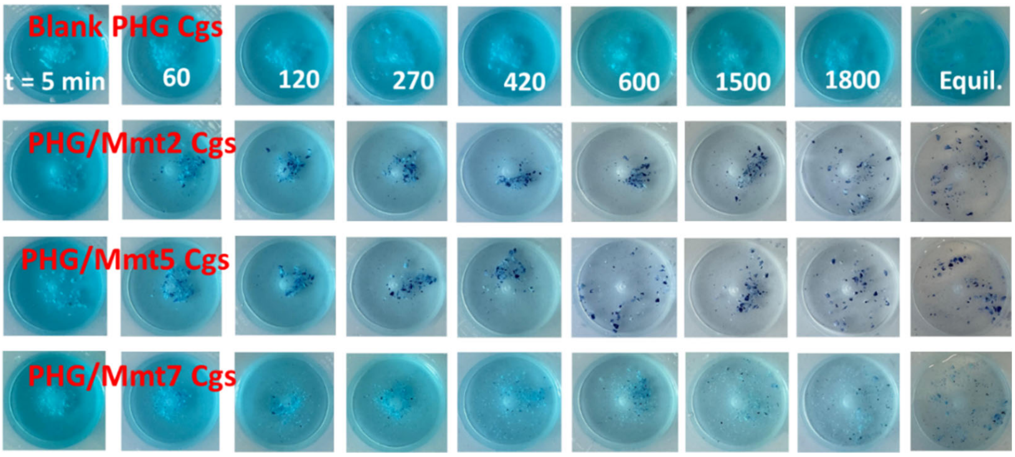

Figure S7. Optical views of time-dependent adsorption of blank and hybrid PHG/Mmt cryogels containing various amount of Mmt in the feed. (Fixed experimental conditions: dye concentration: 10 mg/L, volume of solution: 10 mL, adsorbent mass: 10 mg, contact time: 8 h, pH: 10, stirring speed: 140 rpm and T: 22.5°C).

Table S2. The equations used for pseudo-first-order, pseudo-second-order, Elovich, Avrami kinetic, and intra-particle model for total MB adsorption onto hybrid PHG/Mmt gels.

	Adsorption Kinetic Model	Linearized Equation	Non-linearized Equation	Kinetic parameters	Ref.
Eq.(S1)	Pseudo-first-order	$\ln(q_e - q_t) = \ln q_e - k_1 t$	$q_t = q_e(1 - e^{-k_1 t})$	k_1 is pseudo-first-order rate constant (min^{-1})	[1]
Eq.(S2)	Pseudo-second-order	$\frac{t}{q_t} = \frac{t}{q_e} + \frac{1}{k_2 q_e^2}$	$q_t = \frac{k_2 q_e^2 t}{1 + k_2 q_e t}$	k_2 is pseudo-second-order rate constant (g mg^{-1} min^{-1})	[2]
Eq.(S3)	Avrami	$\ln\left[\ln\left(\frac{q_e}{q_e - q_t}\right)\right] = n_{Av} \ln k_{Av} + n_{Av} \ln t$	$q_t = q_e\left[1 - e^{\left[-(k_{Av}\ t)^{n_{Av}}\right]}\right]$	k_{Av} is Avrami kinetic constant, and n_{Av} is Avrami exponent	[3]
Eq.(S4)	Elovich	$q_t = \frac{1}{\beta}\ln t + \frac{1}{\beta}\ln(\alpha\beta)$	$q_t = \frac{1}{\beta}\ln(\alpha\beta t)$	α is a constant for rate of chemisorption, is a constant for extent of surface coverage of adsorbent	[4]
Eq.(S5)	Intraparticle diffusion model	$q_t = k_{diff} t^{1/2} + C$	$q_t = k_{diff} t^{1/2} + C$	k_{diff} is rate constant for intraparticle diffusion (mg g^{-1} min$^{-1/2}$), and C is a constant for thickness of boundary layer	[5]

Table S3. Thermodynamic parameters for the adsorption of MB dye and adsorption capacity of hybrid PHG/Mmt cryogels calculated from nonlinearized kinetic models.

Hybrid PHG/Mmt cryogels				
Sample	Exp. q_e (mg/g)	PFO model q_e (mg/g)	PSO model q_e (mg/g)	ΔG^o (kJ/mol K)
Blank PHG	0.1450	0.1409	0.1459	8.6758
PHG/Mmt2	2.5025	2.3383	2.5498	-4.5546
PHG/Mmt5	2.6036	2.4099	2.6247	-7.0742
PHG/Mmt7	1.8326	2.1256	1.8570	-3.9867

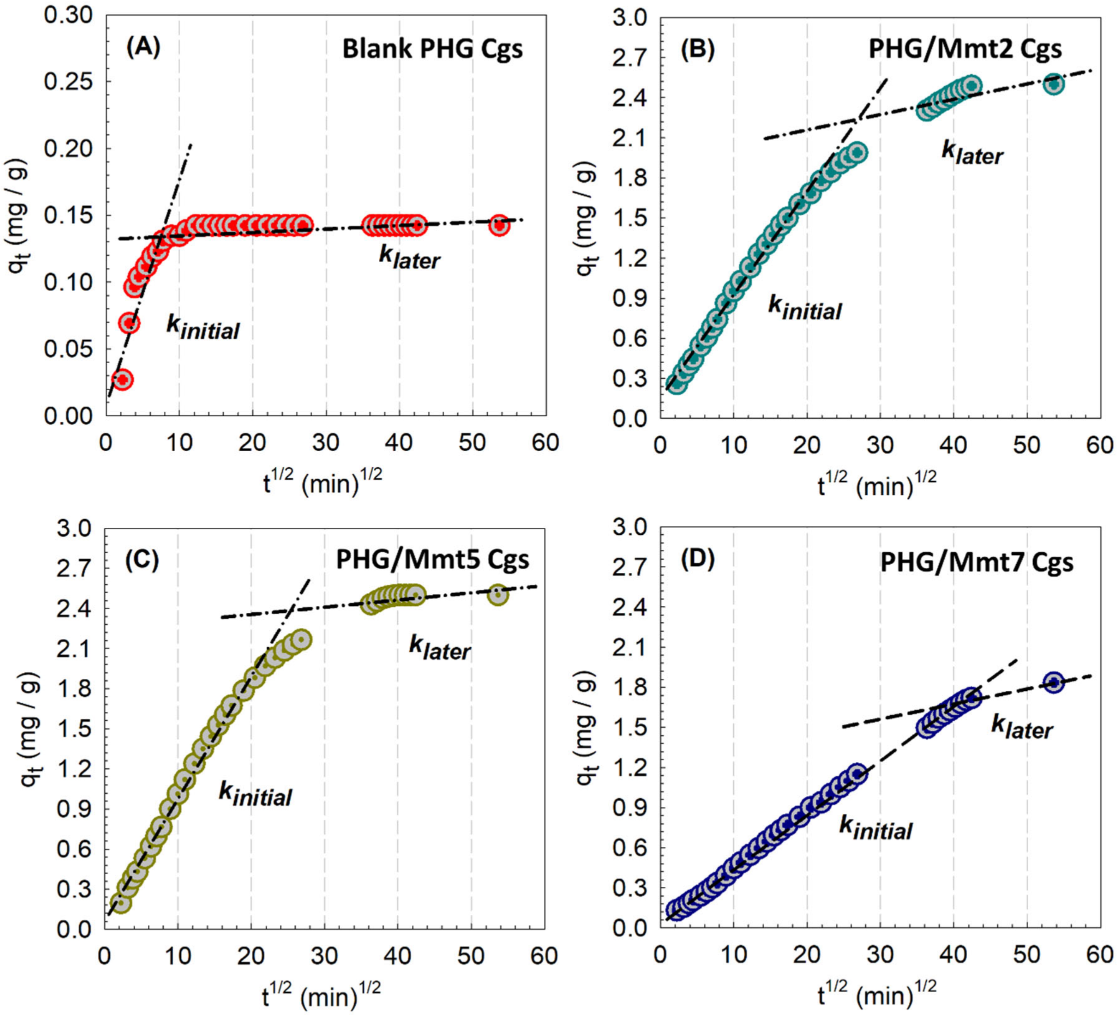

Figure S8. Adjustment of the intraparticle diffusion model by linear fitting of the experimental data.

Table S4. Linear and non-linearized forms of the isotherm models applied in the adsorption of MB dye.

	Types of Isotherm Model	Non-linear Equation	Linear Equation	Description of Isotherm	Ref.
Eq.(S6)	Langmuir	$q_e = \frac{q_{max} K_L C_e}{1+K_L C_e}$	$\frac{C_e}{q_e} = \frac{1}{q_{max} K_L} + \frac{C_e}{q_{max}}$ and $R_L = \frac{1}{1+K_L C_o}$	q_{max} (mg g^{-1}) is maximum adsorption capacity of adsorbent, K_L (L/mg) is Langmuir adsorption constant, R_L is separation factor	[6]
Eq.(S7)	Freundlich	$q_e = K_F C_e^{1/n_F}$	$\ln q_e = \ln K_F + (1/n) 1$	K_F (mg/g)(mg/L)$^{-1/n}$ is Freundlich isotherm constant and n_F is adsorption intensity	[7]
Eq.(S8)	Redlich-Peterson (R-P) isotherm	$q_e = \frac{K_{RP} C_e}{(1+\alpha_{RP} C_e^{\beta_{RP}})}$		K_{RP} (L/g) is Redlich–Peterson isotherm constant, α_{RP} (mg/L) is Redlich–Peterson model constant and β_{RP} are Redlich-Peterson model exponent	[8]
Eq.(S9)	Sips isotherm	$q_e = \frac{K_S C_e^{n_S}}{(1+\alpha_S C_e^{n_S})}$		K_S is Sips equilibrium constant (L mg^{-1}), α_s is Sips isotherm model constant, n_S is Sips model exponent.	[9]
Eq.(S10)	Toth isotherm	$q_e = \frac{q_{max} K_T C_e}{\left[1+(K_T C_e)^{n_T}\right]^{1/n_t}}$		n_T is non-uniform adsorption parameters (between 0 and 1), and K_T is Toth equation parameters.	[10]

References

[1] Lagergren, S. About the Theory of So-Called Adsorption of Soluble Substances. K. Sven. Vetenskapsakad. Handl. **1898**, *24*, 1–39.
[2] Ho, Y.; McKay, G. Pseudo-second order model for sorption processes. *Process. Biochem.* **1999**, *34*, 451–465.
[3] Avrami, M. Kinetics of phase change. II Transformation-time relations for random distribution of nuclei. *J. Chem. Phys.* **1940**, *8*, 212-224. (10.1063/1.1750631).
[4] C. Aharoni, C.; F.C. Tompkins, F.C. Kinetics of adsorption and desorption and the Elovich equation. D.D. Eley, H. Pines, P.B. Weisz (Eds.), Advances in Catalysis and Related Subjects, vol. 21, Academic Press, New York (1970), 1-49.
[5] Weber, W.J.; Morris, J.C. Kinetics of adsorption on carbon from solution. *ASCE Sanit. Eng. Div. J.*, **1963**, *82*, 31-59.

[6] Langmuir I., The adsorption of gases on plane surfaces of glass, mica and platinum*, JACS,* **1918**, *40*, 1361-1403.
[7] Freundlich H., Over the adsorption in solution, J. Phys. Chem., 1906, 57,1100–1107.
[8] Redlich, O.; Peterson, D.L. A Useful Adsorption Isotherm. *Journal of Physical Chemistry*, **1959**, *63*, 1024-1024. http://dx.doi.org/10.1021/j150576a611
[9] Sips, R. On the Structure of a Catalyst Surface. *J. Chem. Phys.* **1948**, *16*, 490–495. DOI:10.1063/1.1746922
[10] Tóth, J. (Editor): Adsorption: Theory, Modeling, and Analysis. Marcel Dekker, Inc., New York-Basel, 2002.
